# High-yielding Pd_2_(dba)_3_·C_6_H_6_-based four-fold Sonogashira coupling with selenophene-conjugated magnesium tetraethynylporphyrin for organic solar cells†

**DOI:** 10.1039/c9ra07393k

**Published:** 2019-10-11

**Authors:** Huan Wang, Takafumi Nakagawa, Meng-Meng Zhang, Keisuke Ogumi, Shangfeng Yang, Yutaka Matsuo

**Affiliations:** Hefei National Laboratory for Physical Science at the Microscale, University of Science and Technology of China Hefei Anhui 230026 China; Department of Mechanical Engineering, School of Engineering, The University of Tokyo 7-3-1 Hongo, Bunkyo-ku Tokyo 113-8656 Japan; Department of Materials Science and Engineering, CAS Key Laboratory of Materials for Energy Conversion, University of Science and Technology of China Hefei Anhui 230026 China; Tokyo Metropolitan Industrial Technology Research Institute 2-4-10 Aomi, Koto-ku Tokyo 135-0064 Japan; Institute of Materials Innovation, Institutes for Innovation for Future Society, Nagoya University Furo-cho, Chikusa-ku Nagoya 464-8603 Japan

## Abstract

A catalytic system using Pd_2_(dba)_3_·(C_6_H_6_)/PPh_3_/CuI for Sonogashira coupling was demonstrated to synthesize a selenophene-conjugated magnesium tetraethynylporphyrin Mg-TEP-(Se-DPP)_4_ (2a). The catalytic system enabled four-fold cross-coupling of the four terminal alkynes of magnesium tetraethynylporphyrin with bromoselenophene-tethered diketopyrrolopyrroles (DPPs) to produce the desired star-shaped 2a in 80% yield. This molecule shows higher solubility in organic solvents, more efficient visible and near-infrared region absorption, and a narrower band gap compared with reference thiophene-conjugated congeners. Two strategies, namely, selenium substitution and end-capping, were investigated to optimize bulk heterojunction structures in the active layers of organic solar cells. The optimized device based on 2a:PC_61_BM exhibited the highest PCE of 6.09% among the tested devices after solvent vapor annealing, owing to efficient exciton dissociation, balanced carrier mobility, and suppressed carrier recombination in the film's ordered morphology.

## Abstract 2

A catalytic system using Pd_2_(dba)_3_·(C_6_H_6_)/PPh_3_/CuI for Sonogashira coupling was demonstrated to synthesize a selenophene-conjugated magnesium tetraethynylporphyrin Mg-TEP-(Se-DPP)_4_ (2a).

## Graphical abstract

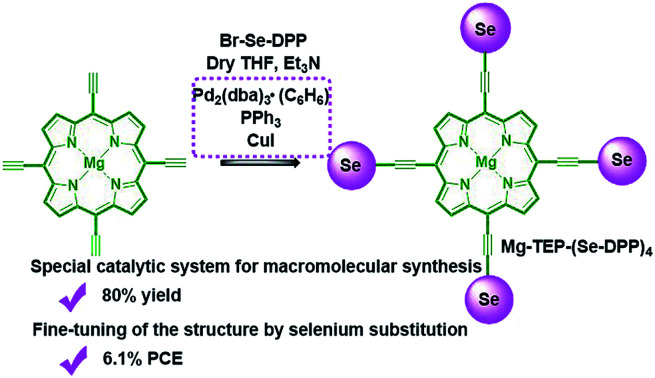

## Introduction

Solution-processed small-molecule (SM) bulk-heterojunction (BHJ) organic solar cells (OSCs) have attracted much greater attention in the past several years due to the revolutionary improvements seen in their power conversion efficiency (PCE).^1–6^ To date, PCEs when using SMs have exceeded 9–14% in single-junction BHJ OSCs as a result of efforts in material innovation and device optimization.^7–17^ Among the useful donor (D)–acceptor (A) materials, porphyrins with a structure of D–(π–A)_2_ and D–(π–A)_4_ conjugated with electron-deficient groups at the *meso*-positions *via* ethynyl bridges exhibit outstanding performance.^18–30^ Active layer materials with these types of porphyrins have some or all of the following advantages: a narrow band gap; a planar configuration contributing to balanced and high carrier mobility; broad absorption in the visible and near-infrared regions; and effective post-treatment processing. In 2013–2016, Peng and co-workers achieved impressive PCEs in excess of 7–9%, with prospects for further improvement, by the strategy of constructing a series of molecules based on a Zn-porphyrin core with two diketopyrrolopyrrole (DPP) units as end groups and employing a D–(π–A)_2_ structure.^18–20^ Our group has focused on the star-shaped D–(π–A)_4_ structure to maximize the extent of conjugation and realize the following advantages: strong, broad absorption in the visible and NIR regions; a narrow band gap; favorable intermolecular interactions; and high carrier mobility.^25,30^ Moreover, magnesium porphyrins have higher solubility than analogous zinc porphyrins because the central Mg atom more readily coordinates with solvent molecules.^25,30^ However, we have also encountered some shortcomings in this design strategy. For example, the extensive conjugation and large molecular geometry tend to result in excessive rigidity, leading to poor solubility, which is unfavorable for device fabrication and synthesis procedures.^30^ A narrow band gap (low energy loss) and broad absorption (high short-circuit current density, *J*_SC_) can be easily achieved by the strategy of increased intramolecular charge transfer with the D–(π–A)_2_ and D–(π–A)_4_ structures, but this alone does not guarantee high PCEs because there could still be energy level mismatch between the HOMO of the electron-donor material and the LUMO of the electron-acceptor material.^30^ To obtain high open-circuit voltage (*V*_OC_), energy level matching and as high a HOMO level of donor material as possible are essential.^31,32^ Accordingly, the current trend in materials development is to maintain certain inherent advantages while avoid certain disadvantages in the future.

To date, great efforts have in materials design have successfully improved PCEs by solving some inherent problems. Selenium substitution is a representative example of a strategy to help reduce the band gap and achieve enhanced and balanced mobility based on fine-tuning of molecular structure in polymer OPVs and organic field-effect transistors.^31,33–36^ However, to our knowledge, the effects of selenium substitution in porphyrin materials have rarely been investigated in recent years even though this approach could provide new insights into the molecular design of OSCs. At the same time, morphological control—particularly achieving small-scale phase separation—is crucial in order to reduce charge recombination and increase charge separation,^37–39^ and selenium substitution could provide the key to unlock further optimization of morphology through post-treatments.^40–49^ Peng and co-workers introduced two selenophene-flanked DPP (Se-DPP) units as end groups on Zn-porphyrin to realize a donor material with moderate PCE of 5.81% in 2016, but it is worth noting that this Se-substituted molecule show wider absorption and a narrower band gap compared with its S analogue.^50^ More recently, Sharma and Langa *et al.* reported a new D–π–A–π–D porphyrin-based SM using selenophene instead of thiophene in the π-bridges and demonstrated a superior PCE of 9.24%.^51^ Peng *et al.* constructed a benzo[1,2-*b*:4,5-*b*′]diselenophene-fused (BDSePhCl) non-fullerene acceptor to achieve an excellent PCE of 13.68% in 2019.^52^ Notably, the blended films of BDSePhCl and polymer donor materials had more suitable phase separation, better charge generation properties, and more balanced carrier mobilities.

When used as acceptor in D–A systems, DPP units are often end-capped with alkyl-thiophenes *via* single bonds.^32,53–59^ Therefore, it is reasonable to introduce extra alkyl-thiophenes into D–(π–A)_2_ and D–(π–A)_4_ structures to convert them into D–(π–A–Ar)_2_ and D–(π–A–Ar)_4_ structures. These new structures have the following advantages: (a) significantly improved solubility, ease of synthesis and separation, and a wider range of thick film thicknesses possible in device optimization,^57–60^ and (b) enhanced light-harvesting, leading to broad absorption especially in the near-infrared region.^53–55,59^ However, a concern is that the alkyl chains of thiophenes might have an undesirable influence on phase separation when there are unfavorable intermolecular interactions in blended films.^60^

**Fig. 1:**
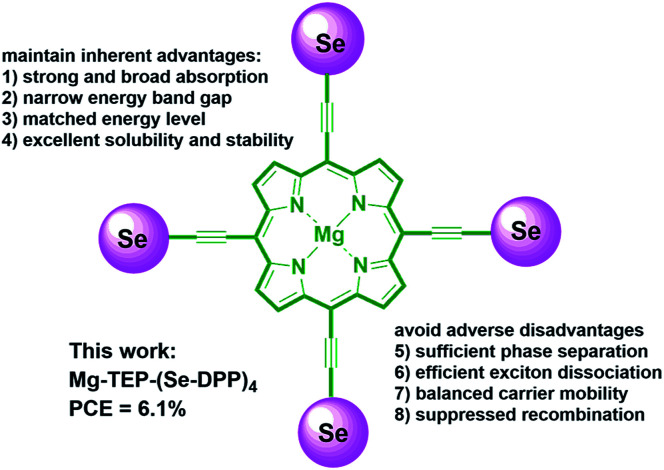
Design concept of Mg-TEPs with four selenophene-flanked DPP units.

**Fig. 2:**
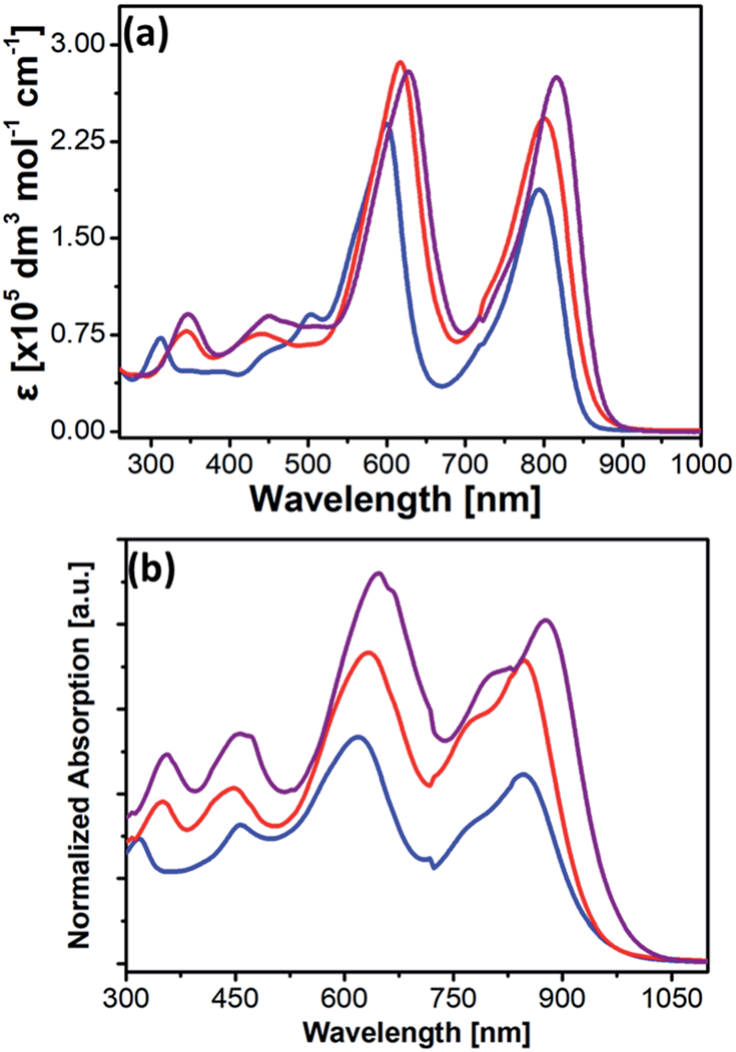
UV-vis absorption spectra of 2a (blue), 2b (red), and 2c (purple) in (a) THF and (b) thin films.

Based on the above considerations and existing challenges, we are interested in systematically exploring the effects of selenium substitution and end-capping with alkyl chains of thiophenes on the photovoltaic performance of SMs with D–(π–A)_4_ and D–(π–A–Ar)_4_ frameworks. In this work, we designed and synthesized three π-conjugated donor molecules based on a Mg-porphyrin core with four Se-DPP units with or without alkyl-thiophenes end-caps, namely, Mg-TEP-(Se-DPP)_4_, Mg-TEP-(S-DPP-Th)_4_ and Mg-TEP-(Se-DPP-Th)_4_ (TEP = magnesium tetraethynyl porphyrin). Importantly, we developed a new catalytic system of Pd_2_(dba)_3_·(C_6_H_6_)/PPh_3_/CuI to effectively suppress porphyrin homocoupling by-products and increase the yield of the desired molecules, such as Mg-TEP-(Se-DPP)_4_ (2a, 80% yield), obtained from Sonogashira coupling. We found that Mg-TEP-(Se-DPP)_4_ (2a) exhibited the following characteristics in comparison with previously reported Mg-TEP-(S-DPP)_4_ (3a), (a) a narrower band gap; (b) more closely matched energy levels, (c) extensive absorption in both the ultraviolet and visible-NIR regions, and (d) slightly poorer morphology of blended films. Moreover, Mg-TEP-(S-DPP-Th)_4_ and Mg-TEP-(Se-DPP-Th)_4_ have excellent solubility. Ultimately, Mg-TEP-(Se-DPP)_4_ showed a decent PCE of 6.09% and photoresponse up to 1000 nm (Fig. 1 and 2).

## Results and discussion

### Synthesis of Mg-TEPs bearing four electron-deficient DPP units

**Scheme 1:**
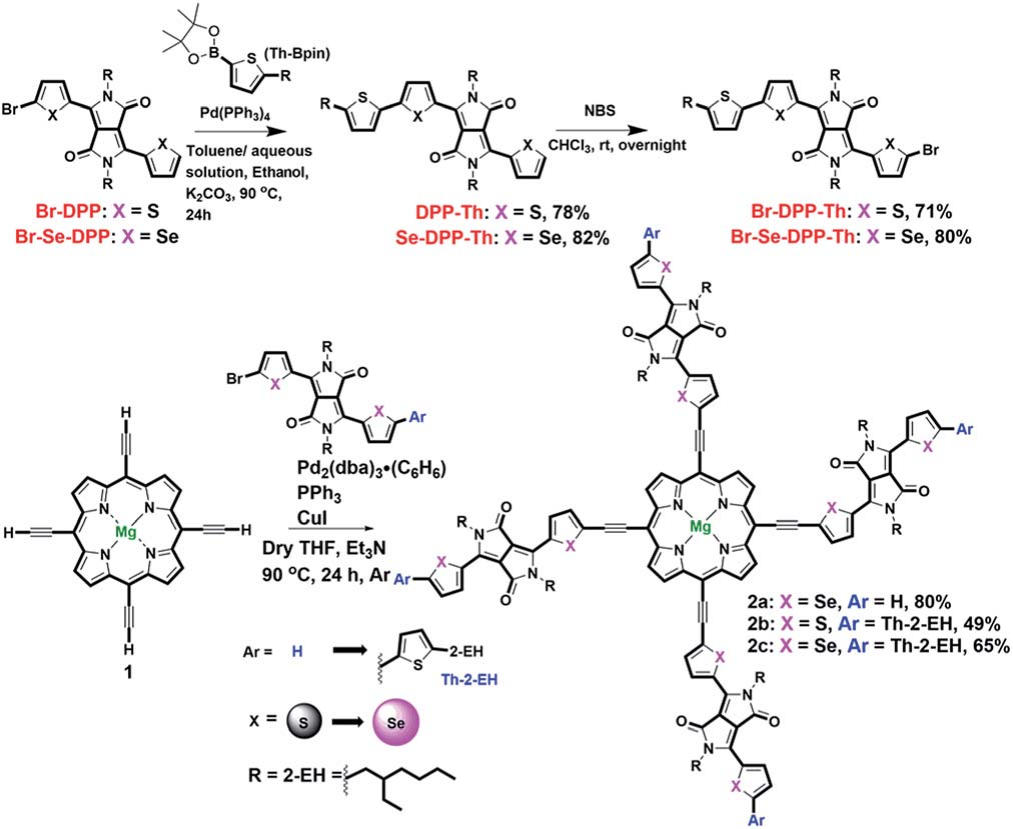
Synthetic route to Mg-TEP-(X-DPP-Ar)_4_.

We synthesized Mg-TEPs conjugated with four electron-deficient DPP units by Sonogashira coupling with monobrominated S-DPP or Se-DPP with or without alkyl-thiophenes as end-caps in different yields (Br-X-DPP-Ar, X = S, Se and Ar = H, Th-2-EH, Scheme 1). We first utilized a stepwise strategy to synthesize the intermediate Mg-TEP-H_4_ (1, magnesium(ii) 5,10,15,20-tetraethynylporphyrin) by our previously reported method.^30,61^ The detailed synthesis procedure is shown in Scheme 1. Here, we also redesigned the synthetic route to Br-DPP-Th and Br-Se-DPP-Th by employing Suzuki coupling instead of Stille coupling to avoid toxic organotin reagents (Schemes 1 and S1†).^57,58^ Then we prepared the desired molecule Mg-TEP-(Se-DPP)_4_ (2a) by Sonogashira coupling with monobrominated Se-DPP. For this reaction, we introduced a new catalytic system of Pd_2_(dba)_3_·(C_6_H_6_)/PPh_3_/CuI to effectively suppress porphyrin homocoupling by-products and increase the yield. Pd_2_(dba)_3_·(C_6_H_6_) was freshly prepared according to previous reports^62–65^ and used immediately, and tetrahydrofuran (THF) and triethylamine were used as solvent and base, respectively. It should be noted that we further used method of freeze–pump–warm for 3 times to remove oxygen as much as possible simultaneously. It is reported that CuI is easily oxidized and leading to form homocoupling and copper porphyrin by-products once trace oxygen exist in reaction systems.^66^ By means of careful preprocessing for reaction systems, we avoided the above problems well. In HRMS spectra of 2a, 2b, 2c of all the field (Fig. S27 and S28†), there were no MS signal for homocoupling products and copper porphyrin. Compounds 2b–c were synthesized by the same procedure as 2a, and 2a–c were purified by silica gel column chromatography and then further purified with preparative gel permeation chromatography (GPC; JAIGEL-2H and JAIGEL-2.5H column, THF). Compounds 2a–c were air-stable black solids.

Compounds 2a–c were highly soluble in common organic solvents such as chloroform, dichloromethane, THF, 1,1,2,2-tetrachloroethane, toluene, 1,2-dichlorobenzene, chlorobenzene, and pyridine, and sparingly soluble in methanol, *n*-hexane, and ethyl acetate. Before silica gel column chromatography, we removed non-porphyrin impurities by washing the compounds with a poor solvent by filtration according to their solubility. Their structures were fully characterized by ^1^H NMR and matrix-assisted laser desorption/ionization time-of-flight (MALDI-TOF) mass spectrometry (Fig. S27–S29†). The chemical structures of 2a–c were confirmed by high-temperature ^1^H NMR spectroscopy using tetrachloroethane-*d*_2_ with 1% pyridine-*d*_5_ at 100 °C (Fig. S11–S13†).

### Photophysical and electrochemical properties

The detailed photophysical and electrochemical properties of 2a, 2b, 2c, and previously reported 3a (Mg-TEP-(S-DPP)_4_) are summarized in Table 1. The absorption spectra of 2a, 2b, and 2c in dilute THF solutions (10^−6^ M) and in thin films from dichloromethane are shown in Fig. 2a and b. The absorption spectra of 2a–c exhibited a strong Soret band around 500–650 nm and a strong CT-band around 700–1000 nm in solution, results that were completely different from those of its precursors Mg-TEPs and DPPs (Fig. S5†). The CT-bands of 2a–c were shifted to the NIR region (700–1000 nm) and showed increased intensity, which is well understood to indicate enhanced intramolecular charge transfer from the Mg-TEPs core to the peripheral DPPs. Compared with previously reported 3a (Table 1), the absorption spectra of 2a–c were red-shifted toward much longer wavelengths and showed much broader absorption ranges both in solution and thin films. For example, two absorption peaks of 2a were observed at 600 and 794 nm in THF. Compared with 2a, the end-capping with alkyl chains of thiophenes in the DPPs of 2b resulted in red-shifted Soret and CT bands (*λ*_max_ = 613 and 799 nm, respectively). Interestingly, the combined effect of both selenium substitution and end-capping with alkyl chains of thiophenes on the DPPs of 2c induced the longest red-shift of these bands (*λ*_max_ = 646 and 853 nm, respectively). In the solid state, the absorption spectra of 2a–c were strongly red-shifted and exhibited panchromatic absorption over a wide range from 400 nm to 1000 nm, which is beneficial for improving *J*_SC_ from the viewpoint of maximum light-harvesting. In comparison with these CT-bands in THF solutions, the maximum absorption peaks for 2a, 2b, and 2c in thin films were red-shifted by 46, 47, and 65 nm, respectively. In addition, all the compounds in thin films show an obvious shoulder peak around 780–800 nm, which may be due to strong intermolecular interactions and aggregation. Based on the onset of the absorption spectrum in thin films, the optical band gaps of 2a, 2b, and 2c were calculated to be 1.35, 1.30, and 1.25 eV.

**Table 1:** Frontier orbital energies of Mg-TEP-(X-DPP-Ar)_4_ in solution as determined by electrochemical measurement and in solids as determined by photoelectron yield spectroscopy

Entry	Film		Solution^a^								Solid^c^
	*λ*_max_ [nm]	*λ*_onset_ [nm]	*λ*_max_ [nm]	*λ*_onset_ [nm]	*E* ^ox^_1/2_ [V]	*E* ^red^_1/2_ [V]	HOMO [eV]	LUMO [eV]	*E*_g_ [eV]	*E*_g_ b [eV]	IP [eV]
2a	620, 840	920	600, 794	866	0.47	−1.11	−5.27	−3.69	1.58	1.35	−5.14
2b	635, 848	950	617, 801	885	0.39	−1.14	−5.19	−3.66	1.53	1.30	−5.18
2c	650, 881	996	628, 816	905	0.32	−1.25	−5.12	−3.55	1.57	1.25	−5.04
3a	606, 826	867	587, 781	847	0.62	−1.01	−5.42	−3.79	1.63	1.43	−5.21

a Values were determined by DPV. Measurements were performed in THF solution containing TBAPF_6_ (0.1 M) as a supporting electrolyte at 25 °C with a scan rate of 100 mV s^−1^. Glassy-carbon, platinum wire, and Ag/AgCl electrodes were used as the working, counter, and reference electrodes, respectively. The potential was measured *versus* Fc/Fc^+^. The HOMO and LUMO levels were estimated by using the following equations: HOMO = −(4.8 + *E*^ox^_1/2_), LUMO = −(4.8 + *E*^red^_1/2_). *E*_g_ = LUMO − HOMO.

b Determined from the absorption onset of the solution, *E*_g_ = 1240/*λ*_onset_ (eV).

c Ionization potential was measured with a RIKEN KEIKI AC-3 photoemission yield spectrometer in air.

**Fig. 3:**
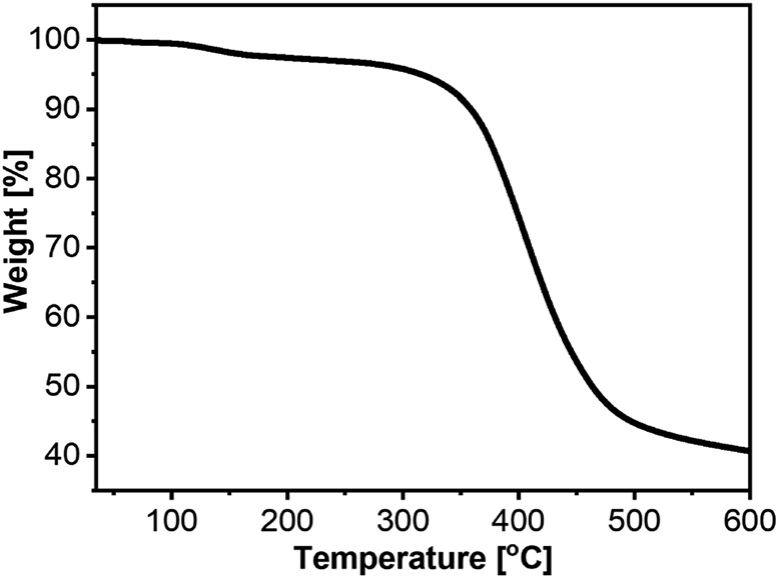
TGA data for 2a under a N_2_ gas flow with a temperature ramp rate of 10 °C min^−1^ up to 600 °C. The temperature with 5% weight loss was 316 °C.

We performed thermogravimetric analysis (TGA) to evaluate whether 2a–c have sufficient thermal stability for further post-treatments in photovoltaic cells. The results showed weight loss of 5% at 316, 328, and 359 °C for 2a, 2b, and 2c, respectively (Fig. 3, S1 and S2†), thus demonstrating their suitability for fabrication of photovoltaic cells.

**Fig. 4:**
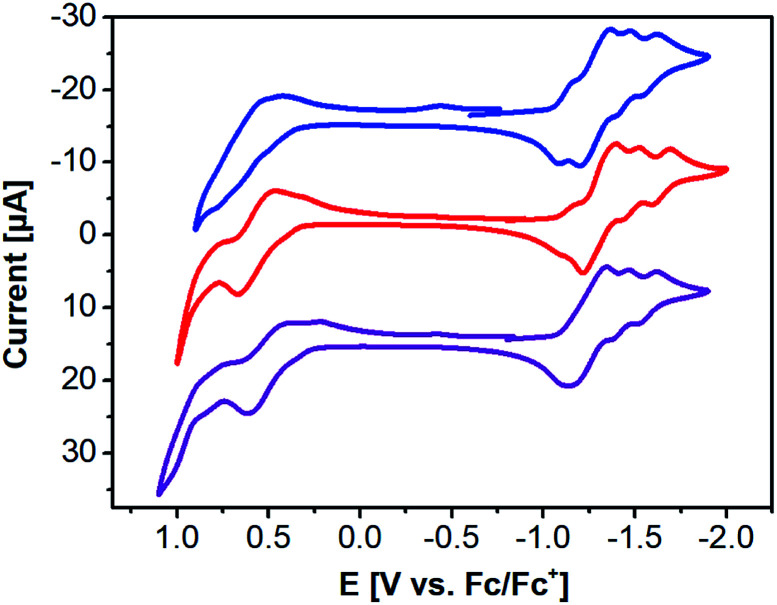
CV of 2a (blue), 2b (red), and 2c (purple) in THF containing TBAPF_6_ (0.1 M) as a supporting electrolyte.

The redox behavior and energy levels of 2a–c were investigated by cyclic voltammetry (CV, Fig. 4) and differential pulse voltammetry (DPV, Fig. S3 and S4†) and the corresponding values are also summarized in Table 1. Compound 2a shows four similar reversible reductions and a broad irreversible oxidation comparable to those of the previously reported 3a. In contrast, a reversible oxidation and an irreversible oxidation as well as three or four reversible reductions were observed for 2b and 2c, respectively. The HOMO and LUMO levels of 2a, 2b, and 2c were determined to be −5.27/−3.69 eV, −5.19/−3.66 eV, and −5.12/−3.55 eV from the DPV results (Table 1). The electrochemical band gaps of 2a, 2b, and 2c were calculated to be 1.58 eV, 1.53 eV, and 1.57 eV, respectively. Compared with 3a, 2a–c all have much narrower electrochemical band gaps. The data in Table 1 show that energy levels and band gaps of 2a–c can be tuned effectively by selenium substitution and end-capping with alkyl chains of thiophenes on the DPPs. It should be noted that the narrowing of the band gap is mainly due to 2a and 2c having higher HOMO level than 3a, since selenium is more polarizable than sulfur because of selenophene having stronger electron-donating ability in comparison with thiophene.^31,67–69^ Interestingly, from the viewpoint of energy level matching, the slightly raised LUMO levels of 2a–c are helpful for increasing the downhill driving force^26,50,70^ (above 0.3 eV) between donor materials 2a–c and PC_61_BM for efficient electron transfer. In addition, we also measured the ionization potential (IP) values for solids of 2a–c in air by photoelectron yield spectroscopy (Table 1): −5.14 eV for 2a, −5.18 eV for 2b, and −5.04 eV for 2c.

### Fabrication of OSCs and photovoltaic properties

**Fig. 5:**
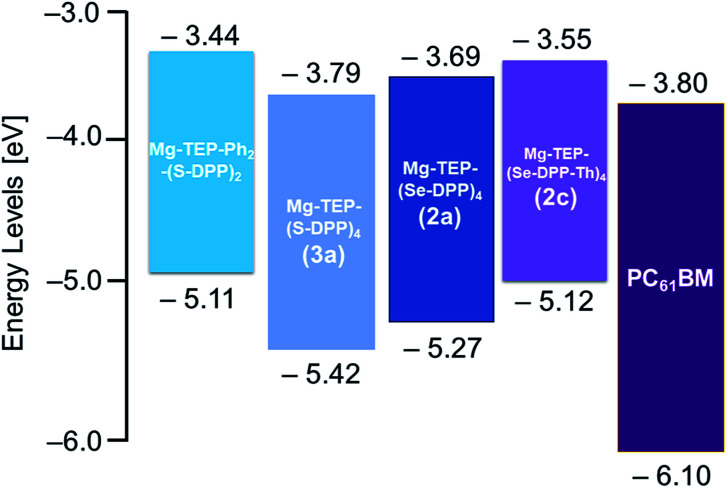
Energy level diagrams for Mg-TEP-Ph_2_-(S-DPP)_2_ (a previously reported DPP_2_ compound, ref. 25), Mg-TEP-(S-DPP)_4_ (a previously reported DPP_4_ compound 3a, ref. 30), 2a, 2c, and PC_61_BM.

To systematically investigate the photovoltaic properties of the porphyrin-based organic electron donor materials, we initially fabricated solution-processed BHJ OSCs with a conventional device structure of ITO/PEDOT:PSS/2a–c:PC_61_BM/LiF/Al (ITO = indium tin oxide; PEDOT:PSS = poly(3,4-ethylenedioxythiophene)polystyrene sulfonate) and tested them under AM 1.5 illumination, 100 mW cm^−2^. The blended films were fabricated by spin-coating a chlorobenzene (CB) solution of 2a, 2b, or 2c and PC_61_BM with a total concentration of 30 mg mL^−1^ (110 nm thickness and mass ratio = 1/1.5). As shown in Table 2, all the as-cast devices exhibited relatively low performance, especially in terms of fill factor (FF) and *J*_SC_. Among these three materials, 2a showed the highest PCE of 4.77% with *V*_OC_ of 0.75 V, *J*_SC_ of 13.84 mA cm^−2^, and FF of 0.463. On the other hand, relatively poor PCEs of less than 2% were obtained for 2b and 2c with lower *J*_SC_ and *V*_OC_. The lower *V*_OC_ of 2b and 2c could partly be ascribed to their slightly higher HOMO levels compared with 2a (Table 1 and Fig. 5). Due to the terminal thiophene alkyl chains, the miscibility between 2b or 2c and PC_61_BM was worse, and we also suspect that excessive intermolecular self-aggregation of 2b or 2c resulted in insufficient phase separation with PC_61_BM in the blended film. Such a blended film is not appropriate for photon absorption, exciton diffusion, and charge transfer because of excessive intermolecular π–π stacking. Ultimately, the unfavorable properties of these blended films lead to lower *J*_SC_, which will be discussed in detail below.

**Table 2:** Photovoltaic performance of the devices under 100 mW cm^−2^ simulated solar irradiation. The devices based on 2b are shown in Table S2. All average values were calculated from more than 8 devices

Entry	Donor	Acceptor	Conc.	SVA [s]	*V*_OC_ [V]	*J*_SC_ [mA cm^−2^]	FF [%]	PCE [%]
**Conventional device structure of ITO/PEDOT:PSS/** **2a** **and** **2c** **:PC**_**61**_ **BM and PC**_**71**_ **BM/LiF/Al**								
1	2a	PC_61_BM	30 mg mL^−1^	—	0.75	13.84	46.30	4.77
2	2a	PC_61_BM	30 mg mL^−1^	THF, 20	0.74	16.70	49.20	6.09
3	2a	PC_71_BM	30 mg mL^−1^	—	0.67	12.09	43.90	3.56
4	2a	PC_71_BM	30 mg mL^−1^	THF, 40	0.68	13.33	41.80	3.74
5	2c	PC_61_BM	30 mg mL^−1^	—	0.56	3.57	51.70	1.02
6	2c	PC_61_BM	30 mg mL^−1^	THF, 30	0.59	5.75	53.10	1.78

**Inverted device structure of ITO/ZnO/** **2a** **and** **2c** **:PC**_**71**_ **BM/MoO**_**3**_ **/Ag**								
1	2a	PC_71_BM	30 mg mL^−1^	—	0.66	8.76	43.34	2.51
2	2a	PC_71_BM	30 mg mL^−1^	CS_2_, 30	0.63	10.34	58.62	3.82
3	2c	PC_71_BM	30 mg mL^−1^	—	0.48	5.23	52.97	1.33
4	2c	PC_71_BM	30 mg mL^−1^	CS_2_, 30	0.54	4.88	55.53	1.46

We attempted to solve the problems of excessive self-aggregation and poor miscibility by employing PC_71_BM as the acceptor and changing the device configuration. It is well known that an inverted configuration is helpful for improving device stability^71–74^ and *J*_SC_.^75^ In a conventional device structure, we were not able to obtain outstanding performance with PC_71_BM, with all devices showing poor or modest efficiency. The device with 2a and PC_71_BM exhibited PCE of 3.56%, which was lower than that of the device using PC_61_BM. The PCE of the device with 2c and PC_71_BM slightly increased to 1.89%. We fabricated inverted devices with a structure of ITO/ZnO/2a–c:PC_71_BM/MoO_3_/Ag. Without any annealing, the device with 2a had PCE of 2.51%. For 2b and 2c, we still only obtained poor PCEs within 1.5%. In other words, these two strategies combined could not effectively solve the inherent problems.

**Fig. 6:**
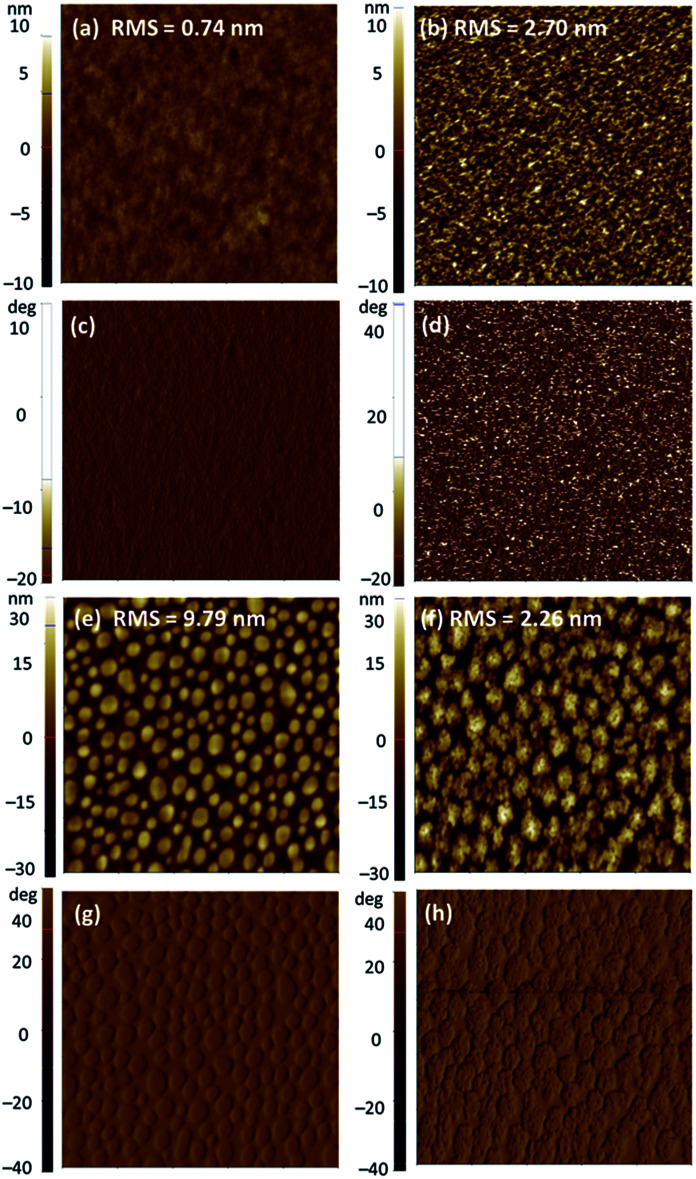
AFM height images (a, b, e, f) and phase images (c, d, g, h) of blended films of 2a:PC_61_BM and 2c:PC_61_BM (1 : 1.5, w/w) as cast (a, c and e, g) and treated with SVA (b, d and f, h). 2a:PC_61_BM as-cast (a and c) and with SVA treatment (b and d); 2c:PC_61_BM as-cast (e and g) and with SVA treatment (f and h).

Subsequently, solvent vapor annealing (SVA) with THF or carbon disulfide (CS_2_) was applied to optimize the blended morphology and increase device efficiency. The 2a-based device showed the highest PCE of 6.09% with slightly reduced *V*_OC_ of 0.74 V, significantly improved *J*_SC_ of 16.70 mA cm^−2^, and similar FF of 0.492 after SVA with THF for 20 s in a conventional configuration. In an inverted device, when SVA treatment with CS_2_ was applied for 30 s, the PCE of the 2a device increased to a relatively high value of 3.82% with effectively improved FF of 0.586 and slightly improved *J*_SC_ of 10.34 mA cm^−2^. By contrast, the performance in both conventional and inverted configurations of the 2b and 2c devices showed limited improvement, despite application of SVA treatment. We concluded that SVA was an effective method to achieve better phase separation for only 2a. To gain insight into the efficiency enhancement due to SVA treatment, the surface morphologies of 2a and 2c were investigated by atomic force microscopy (AFM) over a surface area of 5 μm × 5 μm in tapping mode. As shown in Fig. 6, the AFM height and phase images for the as-cast film of 2a showed a smooth surface with root mean square (RMS) roughness of 0.74 nm without SVA, indicating that 2a already had sufficiently good miscibility with PC_61_BM. After SVA treatment with THF for 20 s, the optimized film of 2a exhibited a slightly rougher surfaces with a slightly increased RMS of 2.70 nm; this case is very similar to previously reported results from several studies.^58,76,77^ We ascribed this to domain growth or well-connected domains for the more ordered morphology of the blended film, which facilitates formation of a finer interpenetrating network to increase the connected interfacial area between the donor and acceptor, which is beneficial for both exciton dissociation and charge transport.^78,79^ As a result, higher *J*_SC_ and FF were obtained for the 2a-based devices. The AFM image of 2c showed a poor morphology with a highly crystalline structure in the blended film. The RMS roughness values of the as-cast film and SVA-treated film were 9.79 nm and 2.26 nm, respectively. Apparently, SVA was not effective enough to reduce such large-scale phase separation. As we suspected, excessive intermolecular self-aggregation of 2c was the main reason for the insufficient phase separation that led to very poor PCEs.

**Fig. 7:**
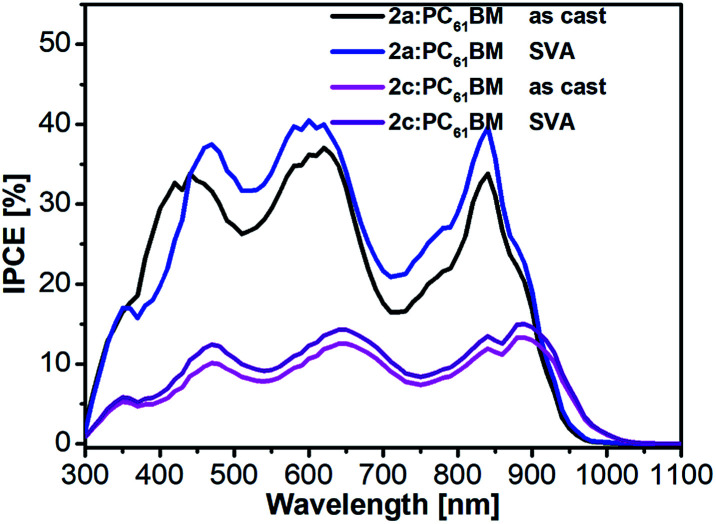
IPCE spectra of as-cast and SVA-treated 2a:PC_61_BM and 2c:PC_61_BM devices in a conventional configuration.

**Fig. 8:**
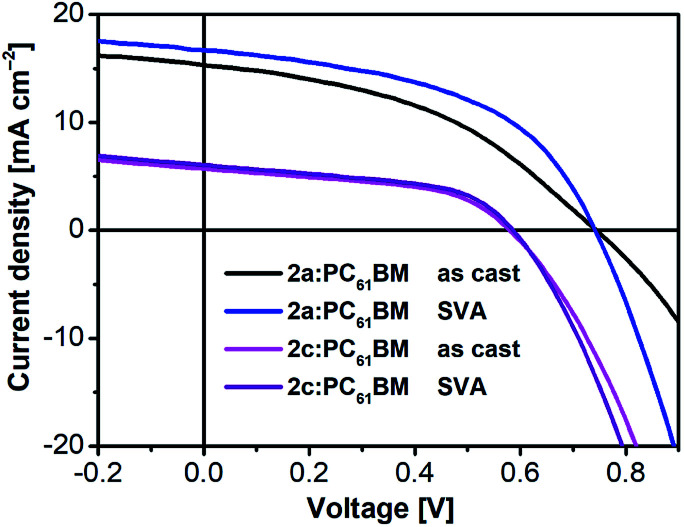
*J*–*V* curves of optimized as-cast and SVA-treated 2a:PC_61_BM and 2c:PC_61_BM devices in a conventional configuration.

To obtain more information about the reason for the enhancement of *J*_SC_ and FF after SVA treatment. We next investigated the incident photon-to-current conversion efficiency (IPCE) spectra (Fig. 7) and external quantum efficiency (EQE) spectra (Fig. S7†) of the as-cast and SVA-treated blended films of 2a and 2c. The *J*–*V* curves of the devices without and with SVA are presented in Fig. 8 and S6† and the detailed photovoltaic parameters are summarized in Tables 2 and S2.† As expected from the absorption spectra, all of the devices exhibited broad IPCE spectra covering the wavelength range from 350 nm to 900 nm and the offset of the IPCE spectra reached 1000 nm. Interestingly, the IPCE values of the 2a-based devices were higher than those of the as-cast and SVA-treated 2c devices across the entire wavelength region, which indicates that the photon-to-electron conversion efficiency of 2a was higher. It also should be noted that the IPCE values for 2a with SVA were slightly higher than those without SVA, which means that SVA had a minor effect on improving IPCE; similar results can also be seen for the EQEs, which are also shown in Fig. S7.†

**Fig. 9:**
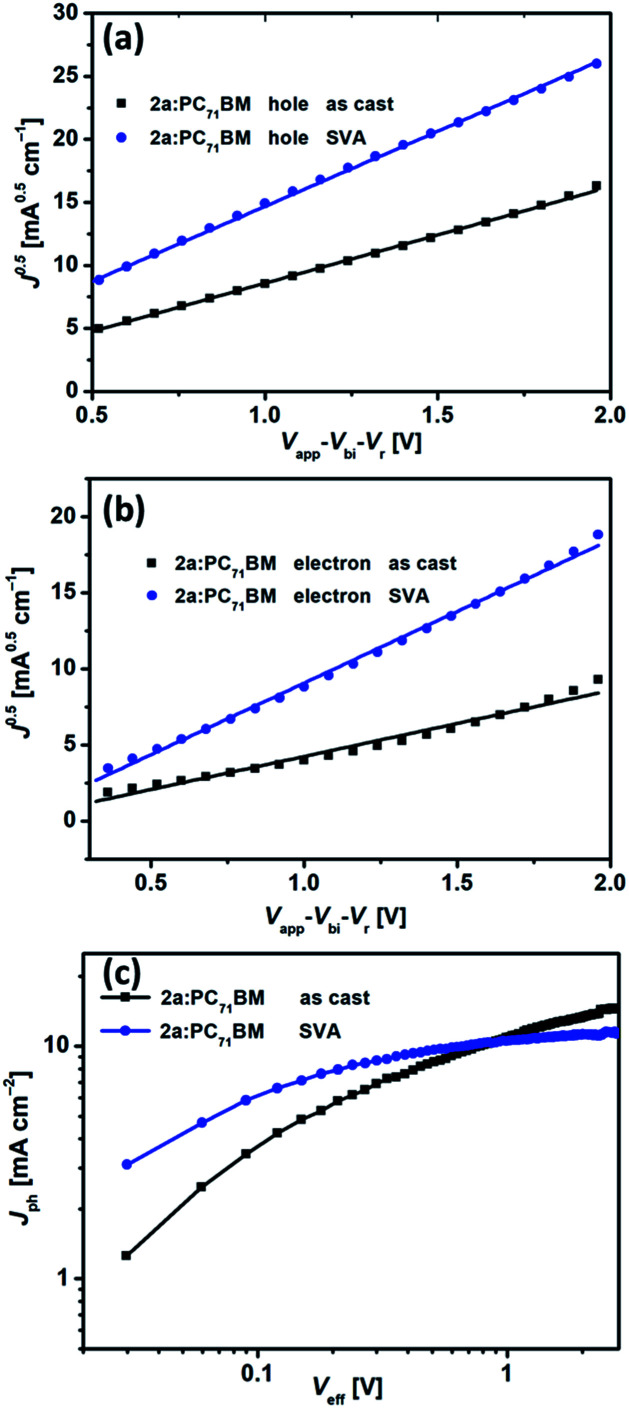
*J* ^0.5^–*V* curves for the (a) hole-only and (b) electron-only devices based on as-cast and SVA-treated 2a:PC_71_BM devices. (c) *J*_ph_*versus V*_eff_ for the optimized as-cast and SVA-treated devices based on 2a:PC_71_BM in an inverted configuration.

To better understand the effect of SVA on charge transport and charge collection, we conducted an in-depth investigation of hole and electron mobilities in bulk heterojunction films of 2a and 2c:PC_71_BM by the space-charge limited current (SCLC) method with almost the same thickness (150 nm). Hole-only and electron-only devices were fabricated with configurations of ITO/PEDOT:PSS/2a or 2c:PC_71_BM/MoO_3_/Ag and ITO/ZnO/2a or 2c:PC_71_BM/Ca/Al, respectively. The *J*–*V* curves for the hole-only and electron-only devices are shown in Fig. S8.† Before SVA, the hole and electron mobilities for the 2a:PC_71_BM devices were 1.68 × 10^−4^ cm^2^ V^−1^ s^−1^ and 0.54 × 10^−4^ cm^2^ V^−1^ s^−1^, respectively, with *μ*_h_/*μ*_e_ of 3.12. After SVA, the hole and electron mobilities for the 2a:PC_71_BM devices increased to 4.08 × 10^−4^ cm^2^ V^−1^ s^−1^ and 2.54 × 10^−4^ cm^2^ V^−1^ s^−1^, respectively, with *μ*_h_/*μ*_e_ of 1.60. For the 2a:PC_71_BM devices, *μ*_e_ improved substantially, while *μ*_h_ only slightly increased. Notably, the *μ*_h_/*μ*_e_ value of 1.60 is closer to 1, indicating more balanced charge transport after SVA treatment (Fig. 9a and b). For as-cast 2c:PC_71_BM devices, *μ*_h_ and *μ*_e_ were 5.41 × 10^−4^ cm^2^ V^−1^ s^−1^ and 0.58 × 10^−4^ cm^2^ V^−1^ s^−1^, respectively, with *μ*_h_/*μ*_e_ of 9.33. The values of *μ*_h_ and *μ*_e_ changed to 5.29 × 10^−4^ cm^2^ V^−1^ s^−1^ and 0.96 × 10^−4^ cm^2^ V^−1^ s^−1^ after SVA treatment, respectively, with *μ*_h_/*μ*_e_ of 5.51. Surprisingly, the *μ*_h_ values of the 2c:PC_71_BM devices both with and without SVA were higher than those of the 2a:PC_71_BM devices, and only *μ*_e_ of the 2c:PC_71_BM was smaller than that of the 2a:PC_71_BM devices after SVA (Fig. S9a and b†), which is consistent with the high crystallinity or aggregation of 2c shown in AFM images. The PCEs of the 2c devices were very poor despite their high mobility. We considered the following disadvantages may account for the low efficiency. (1) Because of high mobility but facile charge recombination as discussed in the introduction, the blended film in the 2c devices showed large-scale phase separation that prevented an adequate interface area for exciton dissociation and resulted in more recombination within the active layer. In short, this situation likely decreased the probability of exciton dissociation. (2) There was unbalanced charge transport.^18,79^ The *μ*_h_/*μ*_e_ value was still 5.51 even after SVA, and the electron mobility was not high. (3) The non-planar configuration of 2c weakened the intermolecular interactions between 2c and the acceptor in the solid film, as did edge-on stacking with the acceptor due to end-capping with alkyl-thiophenes.^80,81^ To verify our speculation about the probability of exciton dissociation in regard to charge generation and charge extraction, we measured the dependence of photocurrent density (*J*_ph_) on the effective voltage (*V*_eff_) in the devices based on the 2a or 2c:PC_71_BM film. The plots of *J*_ph_*versus V*_eff_ are shown in Fig. 9c and S9c,† respectively. In the 2a:PC_71_BM film, *J*_ph_ of both the as-cast and SVA-treated devices increased linearly with increasing *V*_eff_ under low *V*_eff_ conditions up to 0.5 V and reached saturated current densities (*J*_sat_) at *V*_eff_ above 2 V. Such high *V*_eff_ is strong enough for collection of all carriers at the electrodes prior to recombination. The values of *J*_sat_ were 12.08 and 10.99 mA cm^−2^ for the as-cast and SVA-treated 2a devices, respectively. The exciton dissociation probability *P*(*E*,*T*) can be calculated as 78.1% and 94.2% for the as-cast and SVA-treated 2a devices, respectively, under the *J*_SC_ conditions by using the equation *P*(*E*,*T*) = *J*_ph_/*J*_sat_. For the as-cast and SVA-treated 2c devices, *P*(*E*,*T*) can be calculated as 85.0% and 85.1%, respectively. Apparently, SVA was helpful for increasing *P*(*E*,*T*) for both 2a and 2c. Importantly, *P*(*E*,*T*) of 2c with SVA was far less than that of SVA-treated 2a devices, which confirmed our speculation and implies that the 2a-based devices had both more efficient exciton dissociation and more balanced charge transport simultaneously, and together these contributed to the superior performance of these devices.

**Fig. 10:**
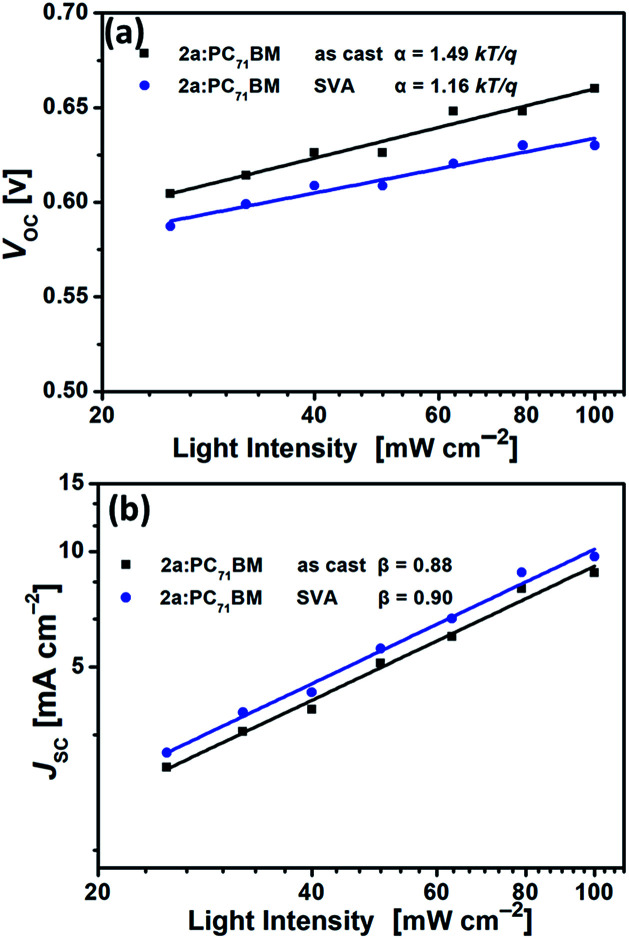
(a) Dependence of *V*_OC_ on light intensity (*P*_light_) for as-cast and SVA-treated 2a:PC_71_BM devices in an inverted configuration. (b) Light intensity (*P*_light_) *versus J*_SC_ for as-cast and SVA-treated 2a:PC_71_BM devices in an inverted configuration.

To further understand the charge recombination behavior of the as-cast and SVA-treated 2a-based devices, the influences of light intensity (*P*_light_) and *V*_OC_ or *J*_SC_ were also investigated. In general, the relationship between *V*_OC_ and light intensity can be described by the formula *V*_OC_ ∝ *α* ln *P*_light_.^78,79^ The primary mechanism is bimolecular recombination when *α* = *kT*/*q* but monomolecular recombination when *α* = 2 *kT*/*q* (*k* is the Boltzmann constant, *T* is the temperature, and *q* is the elementary charge). As shown in Fig. 10a, the as-cast 2a-based device had an *α* value of 1.49*kT*/*q*, while *α* for the SVA-treated 2a-based device was 1.16*kT*/*q*, indicating less monomolecular recombination under open-circuit conditions after SVA treatment. In addition, we further investigated the charge recombination properties by the relationship between *J*_SC_ and light intensity (*P*_light_), which can be described using the index *β* in the formula *J*_SC_ ∝ *P*_light_^*β*^.^82^ When all free carriers are transported to and collected at the electrodes, *β* is equal to 1, which means that bimolecular recombination is almost totally suppressed. The *β* value of less than 1 means that bimolecular recombination occurs to some extent. The *β* values of the 2a-based devices with as-cast and SVA-treated films were 0.88 and 0.90, respectively, indicating that bimolecular recombination was slightly suppressed by SVA treatment. Generally, recombination loss is very closely related to *J*_SC_ and FF;^78,79,82^ thus, SVA helped to improve *J*_SC_ and FF by suppressing carrier recombination in the 2a-based devices (Fig. 10b).

## Conclusion

We demonstrated a new catalytic system using Pd_2_(dba)_3_·(C_6_H_6_)/PPh_3_/CuI in Sonogashira coupling to synthesize a selenophene-conjugated magnesium tetraethynylporphyrins Mg-TEP-(Se-DPP)_4_ (2a) in 80% yield. We designed and synthesized three star-shaped porphyrin-based donor materials (2a, Mg-TEP-(Se-DPP)_4_, 2b, Mg-TEP-(S-DPP-Th)_4_ and 2c, Mg-TEP-(Se-DPP-Th)_4_) with four electron-deficient DPPs with or without alkyl-thiophenes as end-caps. In this work, we applied two strategies (selenium substitution and end-capping with alkyl chains of thiophenes) to optimize the molecular structure with the aim of achieving outstanding performance in photovoltaic device. As we hoped, all three molecules showed broad, strong absorption ranging from 550 and 950 nm, narrow band gaps, and well-matched energy levels with PC_61_BM and PC_71_BM. The optimized devices based on 2a, 2b, and 2c were obtained by SVA treatment and exhibited distinct PCEs of 6.09%, 1.63% and 1.89%, respectively. However, it seems that only selenium substitution played a positive role in improving the PCEs. Compared with 2b and 2c, compound 2a had a more ordered morphology in blended films with higher miscibility and better phase separation with PC_61_BM and PC_71_BM. The highest efficiency of the 2a devices can be ascribed to efficient exciton dissociation, balanced carrier mobility, and suppressed carrier recombination with the more ordered morphology together facilitating achievement of higher *J*_SC_ and FF. By contrast, 2c-based blended films showed poor morphology with high crystallinity and large-scale phase separation, which led to inefficient exciton dissociation and unbalanced carrier mobility, resulting in low efficiency. Even though 2a exhibited the advantages of broader and stronger absorption, a narrower band gap, and more closely matched energy levels, the optimized 2a-based device still exhibited lower efficiency (6.1%) compared with the optimized 3a-based device (7.4%). We attribute this lower efficiency primarily to the slightly poorer morphology of the blended films of 2a compared with 3a, finally, which led to slightly lower *J*_SC_ and FF than those of 3a. We fully recognize that this is a rather pedestrian PCE value among OSCs and further engineering is necessary in the future. Yet, the unsuccessful molecular designs of 2b and 2c also provide insights into the potential adverse effects of the end-capping with alkyl chains of thiophenes and can help researchers avoid such pitfalls in the future. On a positive note, selenium substitution appears to be a promising strategy to develop effective donor materials and high-performance OSCs. In addition, the results of this study highlight the importance of morphological control, particularly achieving suitable phase separation, which is a current trend in device optimization to further improve PCEs.

## Experimental

### [5,10,15,20-tetrakis[3-(Selenophen-2-yl)-2-{2,5-bis(2-ethylhexyl)-6-(selenophen-2-yl)-2,5-dihydropyrrolo[3,4-*c*]pyrrole-1,4-dione-6-yl}-thien-5-ylethynyl]porphyrinato]magnesium(ii) (2a)

A solution of 1 (60.0 mg, 0.13 mmol) in dry THF (35 mL) was added Br-Se-DPP (X = Se, Ar = H, 418 mg, 0.60 mmol), Pd_2_(dba)_3_·C_6_H_6_ (30.0 mg, 30.0 μmol), PPh_3_ (7.8 mg, 30.0 μmol), CuI (2.3 mg, 15.0 μmol), and dry triethylamine (30 mL). After heating at 90 °C for 24 h, the mixture was purified with silica gel column by using CH_2_Cl_2_/CHCl_3_ (20/1) as eluent, and then purified with preparative GPC (JAIGEL-2H and JAIGEL-2.5H column, THF). The solvent was removed under reduce pressure to give the desired product as black powder (301 mg, 80% yield). ^1^H NMR (400 MHz, tetrachloroethane-*d*_2_ with 1% pyridine-*d*_5_, 100 °C): *δ* 9.58 (s, 8H, porphyrin), 8.83 (d, *J* = 4.3 Hz, 4H, selenophene), 8.81–8.76 (m, 4H, selenophene), 8.45 (d, *J* = 5.5 Hz, 4H, selenophene), 8.04 (d, *J* = 4.3 Hz, 4H, selenophene), 7.54 (d, *J* = 5.6 Hz, 4H, selenophene), 4.11 (d, *J* = 7.7 Hz, 8H, NCH_2_), 4.05 (d, *J* = 7.8 Hz, 8H, NCH_2_), 2.11 (s, 4H, CH), 1.97 (s, 4H, CH), 1.52–1.34 (m, 64H, CH_2_), 1.10–0.91 (m, 48H, CH_3_). UV-vis (solution in THF) *λ*_Soret_(*ε*): 600 (2.38 × 10^5^), *λ*_Q_(*ε*): 794 (1.88 × 10^5^). MALDI-TOF-HRMS (+) (*m*/*z*): calcd for C_148_H_164_MgN_12_O_8_Se_8_ (M^+^): 2894.6070, found 2894.6050.

### [5,10,15,20-tetrakis[3-(Thiophen-2-yl)-2-{2,5-bis(2-ethylhexyl)-6-(5′-(2-ethylhexyl)-[2,2′-bithiophen]-5-yl)-2,5-dihydropyrrolo[3,4-*c*]pyrrole-1,4-dione-6-yl}-thien-5-ylethynyl]porphyrinato]magnesium(ii) (2b)

A solution of 1 (60.0 mg, 0.13 mmol) in dry THF (35 mL) was added Br-DPP-Th (X = S, Ar = Th-2-EH, 479 mg, 0.60 mmol), Pd_2_(dba)_3_·C_6_H_6_ (30.0 mg, 30.0 μmol), PPh_3_ (7.8 mg, 30.0 μmol), CuI (2.3 mg, 15.0 μmol), and dry triethylamine (30 mL). After heating at 90 °C for 24 h, the mixture was purified with silica gel column by using CH_2_Cl_2_/CHCl_3_ (100/1) as eluent, and then purified with preparative GPC (JAIGEL-2H and JAIGEL-2.5H column, THF). The solvent was removed under reduce pressure to give the desired product as black powder (210 mg, 49% yield). ^1^H NMR (400 MHz, tetrachloroethane-*d*_2_ with 1% pyridine-*d*_5_, 100 °C): *δ* 9.38 (s, 8H, porphyrin), 9.03 (d, *J* = 5.4 Hz, 4H, thiophene), 8.91 (d, *J* = 2.5 Hz, 4H, thiophene), 7.87 (d, *J* = 4.4 Hz, 4H, thiophene), 7.26 (d, *J* = 3.8 Hz, 4H, thiophene), 7.19 (d, *J* = 3.5 Hz, 4H, thiophene), 6.79 (d, *J* = 3.2 Hz, 4H, thiophene), 4.16 (m, 16H, NCH_2_), 2.85 (d, *J* = 6.6 Hz, 8H, thiophene–CH_2_), 2.11 (m, 8H, CH), 1.87 (m, 4H, CH), 1.59–1.39 (m, 96H, CH_2_), 1.14–0.95 (m, 72H, CH_3_). UV-vis (solution in THF) *λ*_Soret_(*ε*): 617 (2.86 × 10^5^), *λ*_Q_(*ε*): 801 (2.42 × 10^5^). MALDI-TOF-HRMS (+) (*m*/*z*): calcd for C_196_H_236_MgN_12_O_8_S_12_ (M^+^): 3296.4972, found 3296.4956.

### [5,10,15,20-tetrakis[3-(Selenophen-2-yl)-2-{2,5-bis(2-ethylhexyl)-6-(5′-(2-ethylhexyl)thiophen-2-yl-selenophen-2-yl)-2,5-dihydropyrrolo[3,4-*c*]pyrrole-1,4-dione-6-yl}thien-5-ylethynyl]porphyrinato]magnesium(ii) (2c)

A solution of 1 (60.0 mg, 0.13 mmol) in dry THF (35 mL) was added Br-Se-DPP-Th (X = Se, Ar = Th-2-EH, 536 mg, 0.60 mmol), Pd_2_(dba)_3_·C_6_H_6_ (30.0 mg, 30.0 μmol), PPh_3_ (7.8 mg, 30.0 μmol), CuI (2.3 mg, 15.0 μmol), and dry triethylamine (30 mL). After heating at 90 °C for 24 h, the mixture was purified with silica gel column by using CH_2_Cl_2_/CHCl_3_ (150/1) as eluent, and then purified with preparative GPC (JAIGEL-2H and JAIGEL-2.5H column, THF). The solvent was removed under reduce pressure to give the desired product as black powder (310 mg, 65% yield). ^1^H NMR (400 MHz, tetrachloroethane-*d*_2_ with 1% pyridine-*d*_5_, 100 °C): *δ* 9.35 (s, 8H, porphyrin), 8.88 (d, *J* = 4.2 Hz, 4H, selenophene), 8.74 (d, *J* = 4.0 Hz, 4H, selenophene), 8.03 (d, *J* = 4.1 Hz, 4H, selenophene), 7.36 (d, *J* = 4.0 Hz, 4H, selenophene), 7.16 (d, *J* = 3.4 Hz, 4H, thiophene), 6.77 (d, *J* = 2.6 Hz, 4H, thiophene), 4.11 (m, 16H, NCH_2_), 2.83 (d, *J* = 6.7 Hz, 8H, thiophene–CH_2_), 2.15 (m, 8H, CH), 1.71 (m, 4H, CH), 1.55–1.35 (m, 96H, CH_2_), 1.08–0.96 (m, 72H, CH_3_). UV-vis (solution in THF) *λ*_Soret_(*ε*): 628 (2.79 × 10^5^), *λ*_Q_(*ε*): 816 (2.76 × 10^5^). MALDI-TOF-HRMS (+) (*m*/*z*): calcd for C_196_H_236_MgN_12_O_8_S_4_Se_8_ (M^+^): 3677.0484, found 3677.5509.

### OSC devices fabrications

The patterned ITO substrates were cleaned by sonicating for 15 min in surfactant water, distilled water, acetone, and isopropyl alcohol. The substrates were then dried using a N_2_ gun and subjected to 15 min UV/O_3_ treatment. Next, a filtrated PEDOT:PSS (Clevios PVP Al4083) solution was deposited on the substrate *via* spin-coating (3000 rpm for 30 s) followed by thermal annealing in air for 10 min at 120 °C. These devices were carried to the glovebox and the active layer was deposited in the N_2_ atmosphere. A 30 mg mL^−1^ solution of porphyrin derivatives 2a and PC_61_BM in chlorobenzene with was prepared with a 1 : 1.5 w/w donor/acceptor ratio. The films were prepared by spin-coating at 1000 rpm for 30 s. The thickness of active layer was around 90–130 nm. The substrates were transferred into a vacuum chamber. All devices were deposited LiF (0.6 nm) and then Al (80 nm). The active area (0.04 cm^2^) was defined by the geometric overlap between Al and ITO. For the fabrication of inverted devices, ZnO precursor solution was prepared before the device fabrication. 1 g zinc acetate dehydrate was dissolved in a mixture solution of 2-methoxyethanol (10 mL) and ethanolamine (300 μL) under stirring in 60 °C overnight in air for hydrolysis reaction. The ZnO precursor solution was spin-coated onto the cleaned ITO substrate at 3000 rpm for 30 s, and then heated at 200 °C for 30 min in air to form a ZnO film. These substrates were transferred to the glovebox. After cooling down, the active layer was deposited onto ZnO layer as the same methods mentioned above. Finally, the device was transferred into a vacuum chamber (∼10^−5^ torr), MoO_3_ (∼10 nm) and Ag electrode (∼80 nm) were sequentially deposited thermally atop the active layer.

## Conflicts of interest

There are no conflicts to declare.

## Supplementary Material

RA-009-C9RA07393K-s001
