# Supplementary material for: High-yielding Pd2(dba)3·C6H6-based four-fold Sonogashira coupling with selenophene-conjugated magnesium tetraethynylporphyrin for organic solar cells
Source: RSC Adv. 2019 Oct 11;9(56):32562–72. doi: 10.1039/c9ra07393k (PMC9073152; doi:10.1039/c9ra07393k)
Supplement: RA-009-C9RA07393K-s001 [file RA-009-C9RA07393K-s001.pdf]

Electronic Supplementary Material (ESI) for RSC Advances.
This journal is © The Royal Society of Chemistry 2019

Supplementary Information

High-yielding $Pd_2(dba)_3{\cdot}C_6H_6$-based Four-fold Sonogashira Coupling for Selenophene-conjugated Magnesium Tetraethynylporphyrin for Organic Solar Cells

Huan Wang,[a,§] Takafumi Nakagawa,[b,§] Meng-Meng Zhang,[c,§] Keisuke Ogumi,[d] Shangfeng Yang,*[,a,c] Yutaka Matsuo*[,a,b,e]

[a] *Hefei National Laboratory for Physical Science at the Microscale, University of Science and Technology of China, Hefei, Anhui 230026, China*

[b] *Department of Mechanical Engineering, School of Engineering, The University of Tokyo, 7-3-1 Hongo, Bunkyo-ku, Tokyo 113-8656, Japan*

[c] *Department of Materials Science and Engineering, CAS Key Laboratory of Materials for Energy Conversion, University of Science and Technology of China, Hefei, Anhui 230026, China*

[d] *Tokyo Metropolitan Industrial Technology Research Institute, 2-4-10 Aomi, Koto-ku, Tokyo 135-0064, Japan*

[e] *Institute of Materials Innovation, Institutes for Innovation for Future Society, Nagoya University, Furo-cho, Chikusa-ku, Nagoya 464-8603, Japan*

E-mails: matsuo@photon.t.u-tokyo.ac.jp (YM); sfyang@ustc.edu.cn (SY)

Table of Contents

Experimental Section S3

Thermal Properties of the Porphyrin Derivatives S6

Absorption Spectra and Electrochemical Data S7

Photovoltaic Data S8

^{1}H and ^{13}C NMR Spectra S12

MALDI-TOF HRMS Data S20

1. Experimental Section

1.1. General

Materials were purchased from Tokyo Kasei (TCI) Co., Sigma-Aldrich Inc., and other commercial suppliers and used after appropriate purification. Anhydrous solvents (stabilizer-free) were purchased from Sigma-Aldrich Chemical and J&K Chemical. Compound **3** and **4** were synthesized as according to previous literature.[1–2] Compounds **Br-DPP**, **Br-Se-DPP** were prepared according to previous papers.[2–3] Compounds **DPP-Th**, **Se-DPP-Th, Br-DPP-Th**, **Br-Se-DPP-Th** were synthesized according to previously reported procedures.[4] All reactions dealing with air- or moisture-sensitive compounds were carried out in a dry reaction vessel under nitrogen or argon. All reactions were monitored by thin layer chromatography (TLC, eluent, ethyl acetate/CH_2Cl_2/ Petroleum ether). The NMR spectra were measured on a Bruker US400 for ^{1}H NMR and ^{13}C NMR, respectively, reported in parts per million from tetramethylsilane. High-resolution mass spectra were acquired by MALDI using a time-of-flight mass analyzer on Bruker Ultra exTOF/TOF spectrometer. UV-vis absorption was recorded on Shimadzu UV-3600PLUS. Cyclic voltammetry (CV) and differential pulse voltammetry (DPV) were performed using a HOKUTO DENKO HZ-5000 voltammetric analyzer. All CV measurements were carried out in a one-compartment cell under argon gas, equipped with a glassy-carbon working electrode, a platinum wire counter electrode, and an Ag/Ag^+ reference electrode. The solvent with supporting electrolyte was a 0.1 mol L^{-1} acetonitrile solution of tetrabutylammonium hexafluorophosphate ($TBAPF_6$). Current–voltage (J–V) characteristics were measured using a source meter (Keithley 2400) under sun AM 1.5G simulated sunlight irradiation (100 mW/cm^2) from a solar simulator (EMS-35AAA, Ushio Spax Inc.), which was calibrated using a silicon diode (BS-520BK, Bunkoukeiki).

Mobility Measurements:

Hole-only and electron-only devices were fabricated by using hole-only devices with a configuration of ITO/PEDOT:PSS/**2a** and **2c**:$PC_{71}BM$ and $PC_{61}BM/MoO_3$/Ag and electron-only devices with a configuration of ITO/ZnO/ **2a** and **2c**: $PC_{71}BM$ and $PC_{61}BM$/Ca/Al, respectively. The mobilities were extracted by fitting the current density–voltage curves using space charge limited current (SCLC). The equation is as follows (Figure S11)：

$$J = 9\varepsilon_0\varepsilon_r\mu V^2/8d^3$$

By the plots of $J^{0.5}$ vs V, hole and electron mobilities can be calculated.
Where J is the current, ε_0 is the permittivity of free space, ε_r is the relative permittivity of the material, V is the effective voltage, and d is the thickness of the active layer.

1.2. Synthesis of the Starting Materials

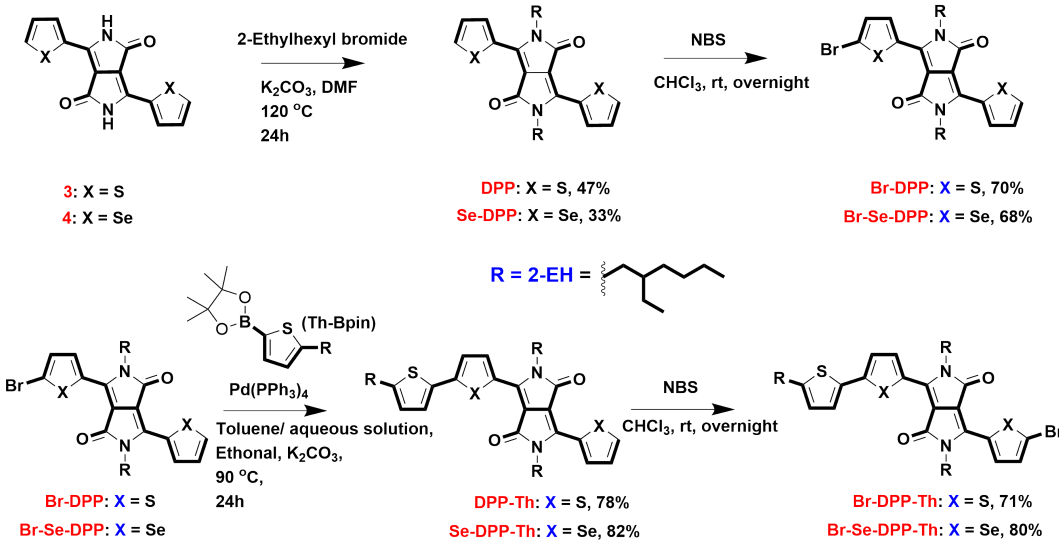

Scheme S1. Synthetic routes of the starting materials.

1.3. Synthetic procedures

2,5-bis(2-ethylhexyl)-3-(5'-(2-ethylhexyl)-[2,2'-bithiophen]-5-yl)-6-(thiophen-2-yl)-2,5-dihydropyrrolo[3,4-c]pyrrole-1,4-dione (DPP-Th). **Th-Bpin** (1.16g, 3.60 mmol), **Br-DPP** (0.72g, 1.20 mmol) were dissolved in to toluene (40 mL), 2 M K_2CO_3 aqueous solution (15 mL), ethanol (5 mL) were added. The mixture was purged with argon for 30 min, then $Pd(PPh_3)_4$ (0.14g, 0.12 mmol) were added. The mixture was stirred at 100 °C for 12h under argon. The mixture was extracted with chloroform (30 mL × 3), washed with water (50 mL × 3) and brine (50 mL × 3), the combined organic layers were dried with anhydrous $MgSO_4$ and concentrated. Finally, the residue was purified with silica gel column by using ethyl acetate/petroleum ether (1/50) to afford compound **DPP-Th** as a purple solid (673 mg, 78% yield). ^{1}H NMR (400 MHz, $CDCl_3$): δ 8.96 (d, J = 4.2 Hz, 2H, thiophene), 8.87 (d, J = 3.9 Hz, 2H, thiophene), 7.29–7.25 (m, 1H, thiophene), 7.24 (d, J = 4.1 Hz, 1H, thiophene), 7.15 (d, J = 3.6 Hz, 1H, thiophene), 6.73 (d, J = 3.6 Hz, 1H, thiophene), 4.04 (m, 4H, N-CH_2), 2.76 (d, J =6.8 Hz, 2H, thiophene-CH_2), 2.00–1.77 (m, 1H, CH), 1.61 (m, 1H, CH), 1.40–1.24 (m, 24H, CH_2), 0.93–0.84 (m, 18H, CH_3). ^{13}C NMR (100 MHz, CDC13): δ 161.92, 161.65, 146.61, 143.78, 140.41, 139.58, 137.18, 135.10, 133.91, 130.30, 130.09, 128.49, 127.42, 126.54, 125.07, 124.09, 108.30, 107.92, 46.02, 41.60, 39.36, 39.21, 34.39, 32.50, 30.48, 30.34, 28.98, 28.68, 28.48, 25.65, 23.80, 23.68, 23.25, 23.20, 23.11, 14.25, 14.20, 14.15, 10.95, 10.68, 10.63.

3-(5-bromothiophen-2-yl)-2,5-bis(2-ethylhexyl)-6-(5'-(2-ethylhexyl)-[2,2'-bithiophen]-5-yl)-2,5-dihydropyrrolo[3,4-c]pyrrole-1,4-dione (Br-DPP-Th). **DPP-Th** (0.86 g, 1.20 mmol) and N-bromosuccinimide (0.22 g, 1.20 mmol) were dissolved in $CHCl_3$ (60 mL), then the solution was protected from light and stirred at room temperature for 24 h. Water (30 mL) was added and the mixture was extracted with chloroform (30 mL × 2). The organic layer was washed with brine (50 mL × 2), and dried over magnesium sulfate. After the solvent was removed under reduced pressure, the crude product was purified by column chromatography on silica gel with petroleum ether/ethyl acetate from 80:1 to 20:1 (v/v) to afford a purple solid (0.68 g, 71% yield). ^{1}H NMR (400 MHz, $CDCl_3$): δ 8.97 (d, J = 4.2 Hz, 1H, thiophene), 8.60 (d, J = 4.2 Hz, 1H, thiophene), 7.23 (d, J = 4.2 Hz, 1H, thiophene), 7.21 (d, J = 4.2 Hz, 1H, thiophene), 7.15 (d, J = 3.6 Hz, 1H, thiophene), 6.73 (d, J = 3.6 Hz, 1H, thiophene), 4.03–3.93 (m, 4H, NCH_2), 2.76 (d, J = 6.7 Hz, 2H, thiophene-CH_2), 1.88 (m, 2H, CH), 1.59 (m, 1H, CH), 1.39–1.22 (m, 24H, CH_2), 0.92–0.85 (m, 18H, CH_3). ^{13}C NMR (101 MHz,

$CDCl_3$): δ161.81, 161.35, 146.78, 144.16, 140.84, 138.04, 137.52, 134.90, 133.87, 131.54, 131.44, 127.30, 126.58, 125.19, 124.12, 118.34, 108.51, 107.75, 46.09, 41.61, 39.35, 39.24, 34.41, 32.51, 30.49, 30.48, 30.32, 30.31, 28.99, 28.68, 28.48, 28.46, 25.66, 23.80, 23.72, 23.70, 23.25, 23.18, 23.12, 14.26, 14.21, 14.16, 10.96, 10.68, 10.63. MALDI-TOF-HRMS (+) (*m*/*z*): calcd for $C_{42}H_{57}BrN_2O_2S_3$ (M^+): 796.2766, found 796.2800.

2,5-bis(2-ethylhexyl)-3,6-di(selenophen-2-yl)-2,5-dihydropyrrolo[3,4-c]pyrrole-1,4-dione (Se-DPP). Potassium carbonate (2.60 g, 18.8 mmol) and 18-crown-6 (~10mg) were added to a solution of **4** (1.66 g, 4.20 mmol) in dry DMF (60 mL), and the mixture was heated at 120 °C for 1 h. Then 2-Ethylhexyl bromide (3.25 g, 16.8 mmol) was injected in one portion and the mixture was stirred for another 24 h at the same temperature. The suspension was then filtered and the solvent removed under reduced pressure. The crude mixture was filtered and washed with methanol to give a copper-color solid, the solid was dried under vacuum and used for next step without further purification (0.86g, 33% yield). ^1H NMR (400 MHz, $CDCl_3$): δ 8.84 (d, J = 4.1Hz, 1H, selenophene), 8.38 (d, J = 5.6 Hz, 1H, selenophene), 7.49 (d, J = 5.6 Hz, 1H, selenophene), 3.97 (m, 2H, N-CH_2), 1.90–1.82 (m, 1H, CH), 1.38–1.20 (m, 16H, CH_2), 0.97–0.83 (m, 12H, CH_3). ^{13}C NMR (100 MHz, $CDCl_3$): δ 161.98, 137.13, 136.70, 134.14, 130.88, 108.14, 45.91, 39.13, 30.39, 30.36, 28.48, 23.72, 23.18, 14.14, 10.61.

3-(5-bromoselenophen-2-yl)-2,5-bis(2-ethylhexyl)-6-(selenophen-2-yl)-2,5 dihydropyrrolo[3,4-c]pyrrole-1,4-dione (Br-Se-DPP). Se-DPP (0.74g, 1.20 mmol) and N-bromosuccinimide (0.22 g, 1.20 mmol) were dissolved in $CHCl_3$ (60 mL), then the solution was protected from light and stirred at room temperature for 24 h. Water (30 mL) was added and the mixture was extracted with chloroform (60 mL × 2). Washed with water (60 mL × 2) and brine (30 mL × 2).The organic layer was separated and dried over magnesium sulfate. After the solvent was removed under reduced pressure, the crude product was purified by column chromatography on silica gel with petroleum ether/dichloromethane/ethyl acetate from 100/5/1 to 20:5/1 (v/v/v) to afford **Br-Se-DPP** as a red powder (0.57 g, 68% yield). ^1H NMR (400 MHz, $CDCl_3$): δ8.86 (d, J = 3.2 Hz, 1H, selenophene), 8.40 (d, J = 3.0 Hz, 2H, selenophene), 7.49 (d, J = 4.1 Hz, 1H, selenophene), 7.40 (d, J = 4.4 Hz, 1H, selenophene), 3.98–3.91 (m, 4H, NCH_2), 1.85 (s, 2H, CH), 1.37–1.21 (m, 16H, CH_2), 0.90–0.83 (m, 12H, CH_3). ^{13}C NMR (100 MHz, $CDCl_3$): δ161.91, 161.76, 142.70, 140.68, 137.52, 137.01, 136.29, 135.57, 134.33, 134.04, 130.98, 123.76, 108.58, 108.00, 46.06, 45.95, 39.12, 30.35, 30.33, 28.44, 23.72, 23.68, 23.18, 14.15, 10.61, 10.59. MALDI-TOF-HRMS (+) (*m*/*z*): calcd for $C_{30}H_{39}BrN_2O_2Se_2$ (M^+): 698.0525, found 698.0532.

2,5-bis(2-ethylhexyl)-3-(5-(5-(2-ethylhexyl)thiophen-2-yl)selenophen-2-yl)-6-(selenophen-2-yl)-2,5-dihydropyrrolo[3,4-c]pyrrole-1,4-dione (Se-DPP-Th). Th-Bpin (1.26 g, 3.90 mmol), compound **Br-Se-DPP** (0.91 g, 1.30 mmol) were dissolved in to (50 mL) toluene and 2 M aqueous K_2CO_3 solution (20 mL), ethanol (8 mL) were added. The mixture was purged with argon for 30 min. Then Pd $(PPh_3)_4$ (0.15 g, 0.13 mmol) were added. The mixture was stirred at 100 °C for 12h under argon. the mixture was extracted with chloroform (60 mL × 2), washed with water (60 mL × 2) and brine (30 mL × 2), the combined organic layers were dried with anhydrous $MgSO_4$ and concentrated. Finally, the residue was purified with silica gel column by using ethyl acetate/petroleum ether (1/60) to afford compound **Se-DPP-Th** as a purple solid (866 mg, 82% yield). ^1H NMR (400 MHz, $CDCl_3$): δ8.82 (d, J = 4.1 Hz, 1H, selenophene), 8.77 (d, J = 4.4 Hz, 1H, selenophene), 8.34 (d, J = 5.6 Hz, 1H, selenophene), 7.46 (d, J = 4.1 Hz, 1H, selenophene), 7.34 (d, J = 4.4 Hz, 1H, selenophene), 7.11 (d, J = 3.6 Hz, 1H, thiophene), 6.70 (d, J = 3.6 Hz, 1H, thiophene), 4.04–3.87 (m, 4H, N-CH_2), 2.74 (d, J = 6.8 Hz, 2H, thiophene-CH_2), 1.88 (m, 2H, CH), 1.59 (m, 1H, CH), 1.36–1.23 (m, 24H, CH_2), 0.92–0.83 (m, 18H, CH_3). ^{13}C NMR (100 MHz, $CDCl_3$): δ162.01, 161.72, 149.78, 146.86, 141.92, 141.34, 137.92, 136.77, 136.39, 136.34, 134.24, 131.34, 130.81, 126.65, 125.99, 125.74, 108.36, 108.07, 46.01, 45.91, 41.54, 39.11, 34.43, 32.48, 30.37, 30.35, 28.96, 28.49, 28.48, 28.46, 25.64, 23.73, 23.17, 23.09, 14.24, 14.15, 14.13, 10.94, 10.62.

3-(5-bromoselenophen-2-yl)-2,5-bis(2-ethylhexyl)-6-(5-(5-(2-ethylhexyl)thiophen-2-yl)selenophen-2-yl)-2,5-dihydropyrrolo[3,4-c]pyrrole-1,4-dione (Br-Se-DPP-Th). Se-DPP-Th

(1.22 g, 1.50 mmol) and N-bromosuccinimide (0.27 g, 1.50 mmol) were dissolved in $CHCl_3$ (60 mL), then the solution was protected from light and stirred at room temperature for 24 h. Water (30 mL) was added and the mixture was extracted with chloroform (30 mL × 2), washed with water (30 mL × 2) and brine (30 mL × 2), and dried over magnesium sulfate. After the solvent was removed under reduced pressure, the crude product was purified by column chromatography on silica gel with dichloromethane/ethyl acetate from 100:1 to 30:1 (v/v) to afford a purple powder (1.07 g, 80% yield). ^{1}H NMR (400 MHz, $CDCl_3$): δ 8.79 (s, 1H, selenophene), 8.37 (s, 1H, selenophene), 7.39 (d, J = 4.3 Hz, 1H, selenophene), 7.35 (d, J = 4.3 Hz, 1H, selenophene), 7.12 (s, 1H, thiophene), 6.71 (d, J = 3.6 Hz, 1H, thiophene), 3.92 (m, 4H, N-CH_2), 2.75 (s, 2H, thiophene-CH_2), 1.95–1.82 (m, 2H, CH), 1.60 (m, 1H, CH), 1.41–1.18 (m, 24H, CH_2), 0.95–0.77 (m, 18H, CH_3). ^{13}C NMR (101 MHz, $CDCl_3$): δ 161.82, 161.37, 150.17, 146.99, 142.19, 138.25, 136.29, 135.15, 134.20, 131.19, 126.67, 126.00, 125.83, 123.32, 108.76, 46.02, 41.52, 39.05, 34.42, 32.46, 30.32, 28.94, 28.45, 28.42, 28.39, 25.61, 23.73, 23.71, 23.68, 23.17, 23.15, 23.08, 14.24, 14.14, 14.12, 10.93, 10.60, 10.58. MALDI-TOF-HRMS (+) (m/z): calcd for $C_{42}H_{57}BrN_2O_2SSe_2$ (M^+): 892.1655, found 892.1653.

2. Thermal Properties of the Porphyrin Derivatives

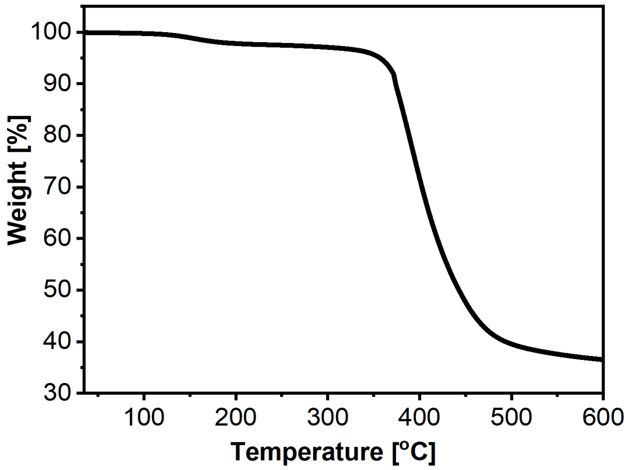

Figure S1. TGA data for **2b** under a N_2 gas flow with temperature ramp rate of 10 °C/min until 600 °C. Temperature with 5% weight loss is 327.89 °C.

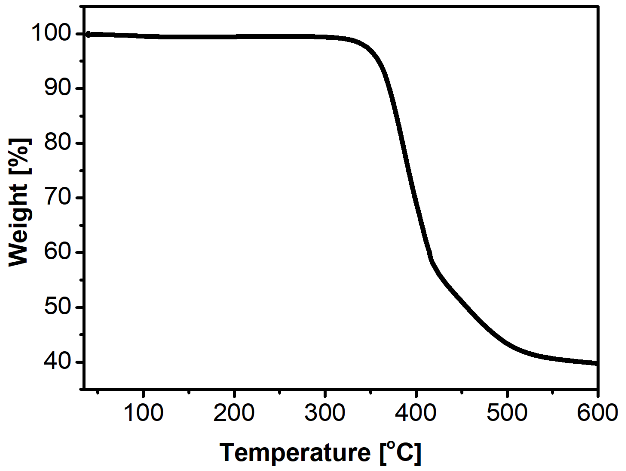

Figure S2. TGA data for **2c** under a N_2 gas flow with temperature ramp rate of 10 °C/min until 600 °C. Temperature with 5% weight loss is 359.30 °C.

3. Absorption Spectra and Electrochemical Data

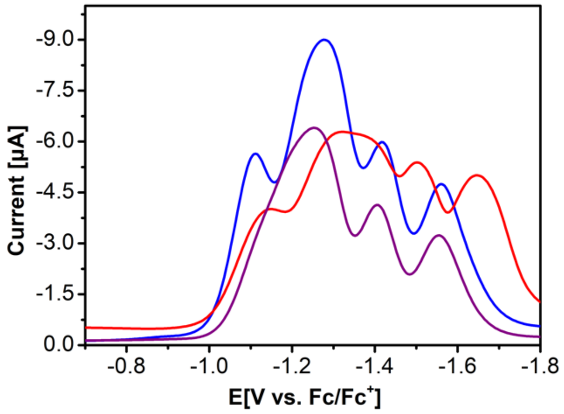

Figure S3. Differential pulse voltammetry (DPV) of the reduction range for **2a** (blue), **2b** (red) and **2c** (purple) in THF.

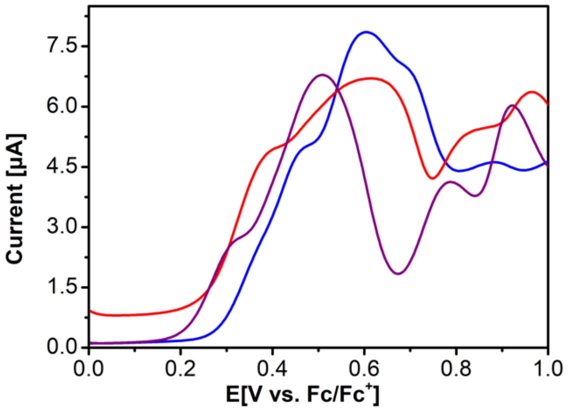

Figure S4. Differential pulse voltammetry (DPV) of the oxidation range for **2a** (blue), **2b** (red) and **2c** (purple) in THF.

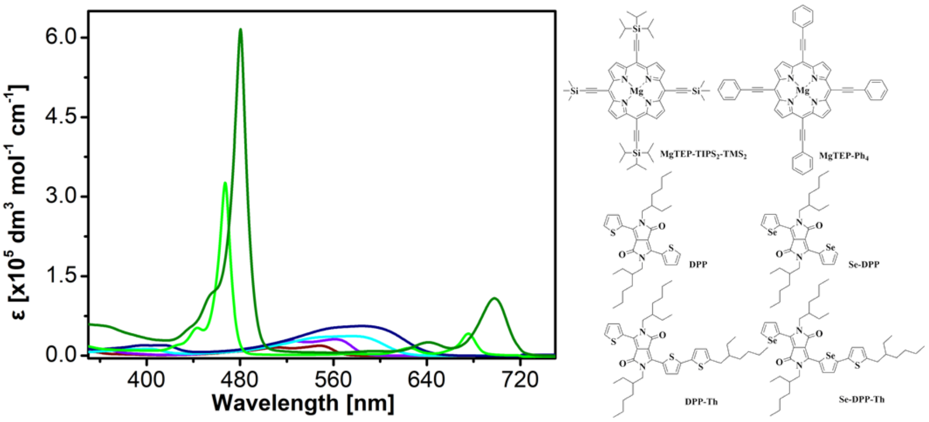

Figure S5. UV-vis absorption spectra of **Mg-TEP-TMS$_2$-TIPS$_2$** (green), **Mg-TEP-Ph$_4$** (olive), **DPP** (wine), **Se-DPP** (violet), **DPP-Th** (cyan) and **Se-DPP-Th** (navy) in THF.

4. Photovoltaic Data

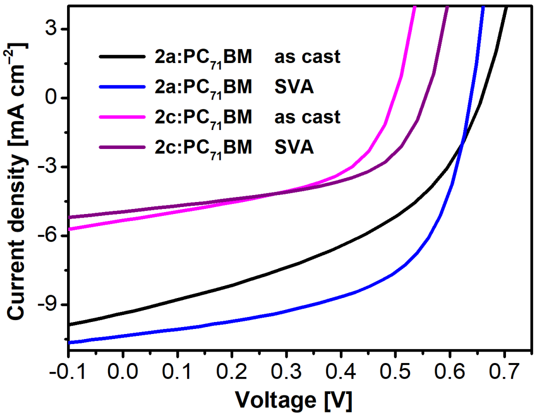

Figure S6. The *J–V* curves of optimized **2a**:$PC_{71}BM$ and **2c**:$PC_{71}BM$ devices without and with SVA treatment in inverted configuration.

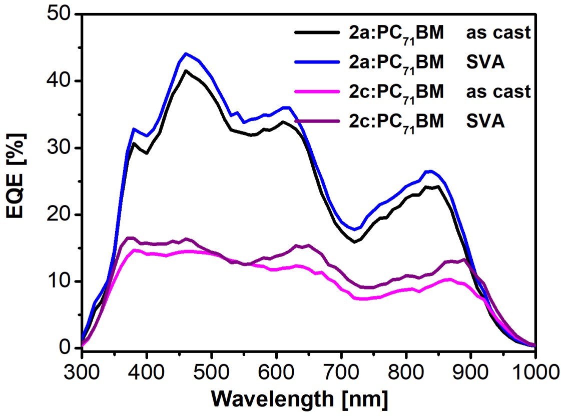

Figure S7. The EQE of optimized devices using as-cast and SVA-treated **2a** and **2c**:$PC_{71}BM$ in inverted configuration.

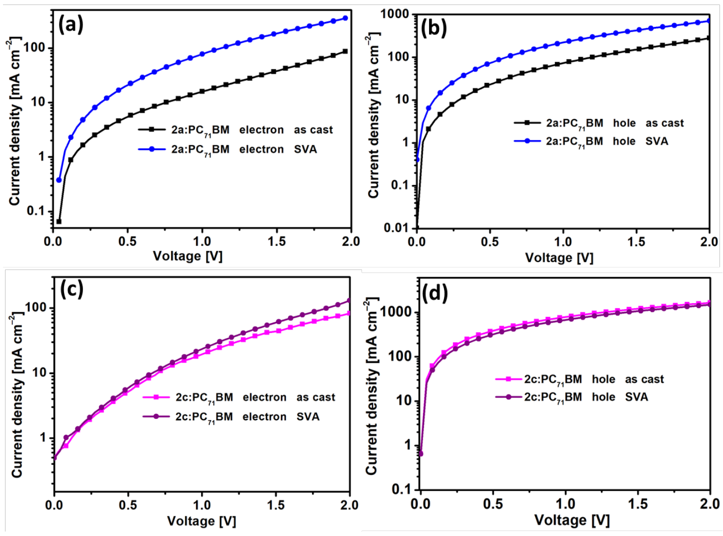

Figure S8. The hole mobilities and electron mobilities of **2a**:$PC_{71}BM$ and **2c**:$PC_{71}BM$ before and after SVA at the optimized device condition.

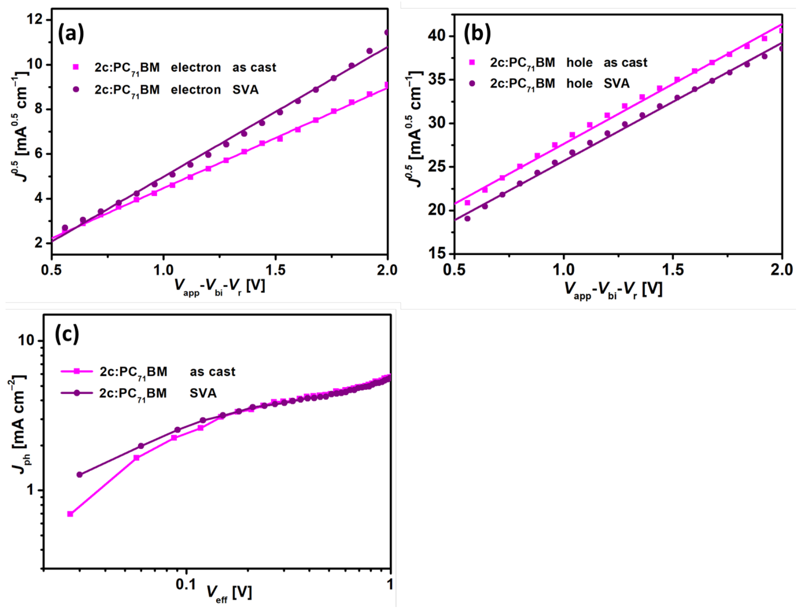

Figure S9. The $J^{0.5}$–V curves for the (a) electron-only and (b) hole-only devices based on as-cast and SVA-treated **2c**:$PC_{71}BM$ devices. (c) J_{ph} versus V_{eff} under the optimized as-cast and SVA-treated devices based on **2c**:$PC_{71}BM$ in inverted configuration.

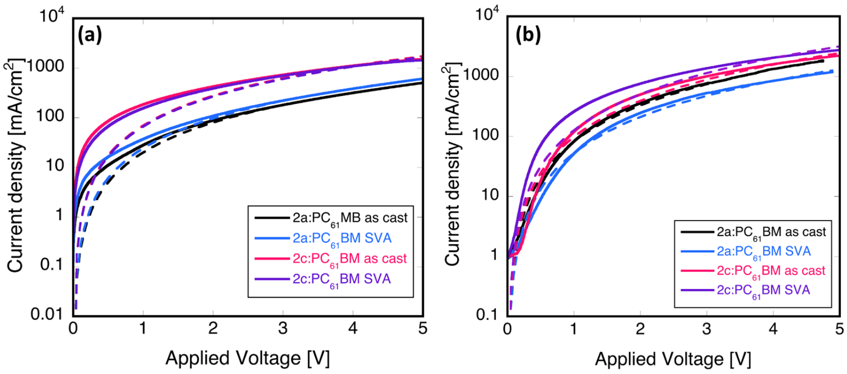

Figure S10. The hole mobilities and electron mobilities of **2a**:$PC_{61}BM$ and **2c**:$PC_{61}BM$ before and after SVA at the optimized device condition measured by SCLC method and the detailed parameters are summarized in **Table S1**.

Table S1. The detailed parameters of the hole mobility and electron mobility of **2a**:$PC_{61}BM$ and **2c**:$PC_{61}BM$ before and after SVA at the optimized device condition.

**2a:PC₆₁BM**	μ_e (cm² V⁻¹ s⁻¹)	μ_h (cm² V⁻¹ s⁻¹)	μ_h/μ_e	**2c:PC₆₁BM**	μ_e (cm² V⁻¹ s⁻¹)	μ_h (cm² V⁻¹ s⁻¹)	μ_h/μ_e
as cast	5.3×10^{-4}	1.3×10^{-4}	0.25	as cast	6.4×10^{-4}	4.5×10^{-4}	0.70
SVA	3.4×10^{-4}	1.5×10^{-4}	0.45	SVA	8.2×10^{-4}	4.4×10^{-4}	0.54

Table S2. Photovoltaic performance of the devices under 100 mW/cm² simulated solar irradiation.

conventional device structure of ITO/PEDOT:PSS/**2b–c**:$PC_{61}BM$ and $PC_{71}BM$ /LiF/Al								
	donor	acceptor	conc	SVA [s]	V_{OC} [V]	J_{SC} [mA cm^{-2}]	FF [%]	PCE [%]
1	**2b**	$PC_{61}BM$	30 mg/mL	–	0.73	4.15	46.30	1.39
2	**2b**	$PC_{61}BM$	30 mg/mL	THF 30	0.73	4.29	49.20	1.55
5	**2c**	$PC_{71}BM$	30 mg/mL	–	0.58	6.30	51.50	1.89
6	**2c**	$PC_{71}BM$	30 mg/mL	THF 30	0.59	4.65	45.00	1.22
inverted device structure of ITO/ZnO/**2b**:$PC_{71}BM/MoO_3$/Ag								
	donor	acceptor	conc	SVA [s]	V_{OC} [V]	J_{SC} [mA cm^{-2}]	FF [%]	PCE [%]
1	**2b**	$PC_{71}BM$	30 mg/mL	–	0.57	5.47	30.66	0.96
2	**2b**	$PC_{71}BM$	30 mg/mL	CS_2 30	0.58	5.53	50.70	1.63

Table S3. Photovoltaic performance of **2a** in BHJ devices with different D/A ratio.

conventional device structure of ITO/PEDOT:PSS/**2a**:$PC_{61}BM$/LiF/Al				
D:A	V_{OC} [V]	J_{SC} [mA cm^{-2}]	FF [%]	PCE [%]
5:4	0.65	13.32	44.46	3.85
1:1	0.72	15.42	46.83	5.20
1:1.5	0.74	16.70	49.20	6.09
1:2	0.76	15.82	44.91	5.40

Table S4. Photovoltaic performance of the **2a** in the BHJ devices with different SVA time.

conventional device structure of ITO/PEDOT:PSS/**2a**:$PC_{61}BM$/LiF/Al				
SVA	V_{OC} [V]	J_{SC} [mA cm^{-2}]	FF [%]	PCE [%]
as-cast	0.75	13.84	46.30	4.77
THF 10s	0.744	15.31	0.420	4.79
THF 20s	0.74	16.70	0.49	6.09
THF 40s	0.74	13.98	0.44	4.59
THF 60s	0.74	16.33	0.50	5.96
THF 80s	0.75	14.69	0.48	5.27

Table S5. Photovoltaic performance of the **2a** in the BHJ devices with different SVA time.

inverted device structure of ITO/ZnO/**2a**:$PC_{71}BM/MoO_3$/Ag				
SVA	V_{OC} [V]	J_{SC} [mA cm^{-2}]	FF [%]	PCE [%]
as-cast	0.65	9.46	43.47	2.69
CS_2 20s	0.65	10.83	44.79	3.14
CS_2 30s	0.63	10.34	58.62	3.82
CS_2 40s	0.63	9.24	58.67	3.39
CS_2 60s	0.60	3.33	43.78	0.88

Table S6. Photovoltaic performance of the devices under 100 mW/cm^2 simulated solar irradiation.

inverted device structure of ITO/ZnO/**2a**:$PC_{71}BM/MoO_3$/Ag					
**2a**:$PC_{71}BM$	J_{SC} [mA cm^{-2}]	J_{SC}^{cal} [mA cm^{-2}]	error	J_{SAT} [mA cm^{-2}]	P(E,T)[a]
as cast	9.46	9.16	–3.17%	12.08	78.3%
SVA	10.34	9.86	–4.46%	10.99	94.1%
inverted device structure of ITO/ZnO/**2c**:$PC_{71}BM/MoO_3$/Ag					
**2c**:$PC_{71}BM$	J_{SC} [mA cm^{-2}]	J_{SC}^{cal} [mA cm^{-2}]	error	J_{SAT} [mA cm^{-2}]	P(E,T)[a]
as cast	5.23	4.44	–15.1%	6.14	85.0%
SVA	4.88	3.79	–22.3%	5.74	85.1%

[a] Exciton dissociation probability under short-circuit condition.

Table S7. Thickness influence on photovoltaic performance of **2a** in BHJ devices after SVA for 20s.

Thickness [nm]	V_{OC} [V]	J_{SC} [mA cm^{-2}]	FF [%]	PCE [%]
90	0.75	15.67	46.71	5.49
110	0.74	16.70	49.20	6.09
130	0.73	16.97	47.39	5.87

7. ^{1}H and ^{13}C NMR Spectra

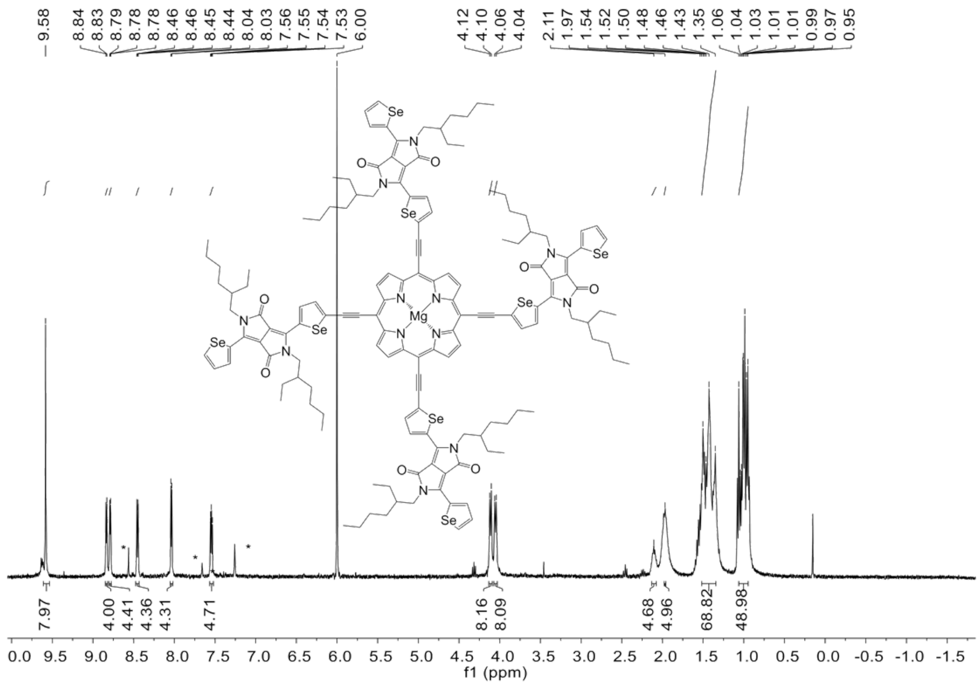

Figure S11. ^{1}H NMR spectrum for **2a**.

^{1}H NMR (400 MHz, tetrachloroethane-d_2 with 1% pyridine-d_5*, 100 °C): δ 9.58 (s, 8H, porphyrin), 8.83 (d, J = 4.3 Hz, 4H, selenophene), 8.81–8.76 (m, 4H, selenophene), 8.45 (d, J = 5.5 Hz, 4H, selenophene), 8.04 (d, J = 4.3 Hz, 4H, selenophene), 7.54 (d, J = 5.6 Hz, 4H, selenophene), 4.11 (d, J = 7.7 Hz, 8H, NCH_2), 4.05 (d, J = 7.8 Hz, 8H, NCH_2), 2.11 (s, 4H, CH), 1.97 (s, 4H, CH), 1.52–1.34 (m, 64H, CH_2), 1.10– 0.91 (m, 48H, CH_3).

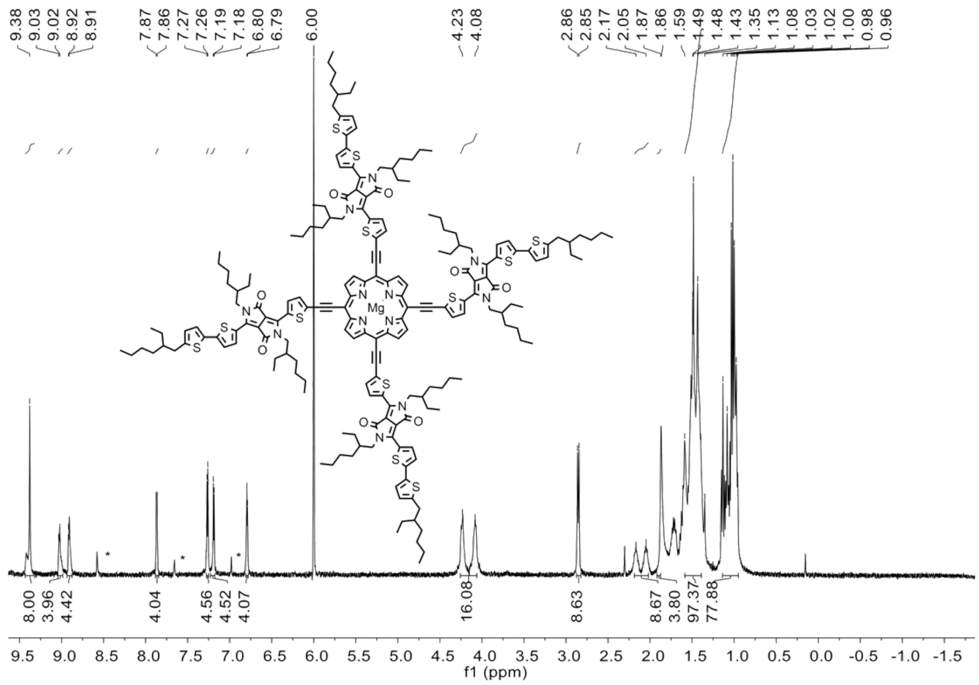

Figure S12. ^{1}H NMR spectrum for **2b**.

^{1}H NMR (400 MHz, tetrachloroethane-d_2 with 1% pyridine-d_5*, 100 °C): δ 9.38 (s, 8H, porphyrin), 9.03 (d, J = 5.4 Hz, 4H, thiophene), 8.91 (d, J = 2.5 Hz, 4H, thiophene), 7.87 (d, J = 4.4 Hz, 4H, thiophene), 7.26 (d, J = 3.8 Hz, 4H, thiophene), 7.19 (d, J = 3.5 Hz, 4H, thiophene), 6.79 (d, J = 3.2 Hz, 4H, thiophene), 4.16 (m, 16H, NCH_2), 2.85 (d, J = 6.6 Hz, 8H, thiophene -CH_2), 2.11 (m, 8H,CH), 1.87 (m, 4H, CH), 1.59–1.39 (m, 96H, CH_2), 1.14–0.95 (m, 72H, CH_3).

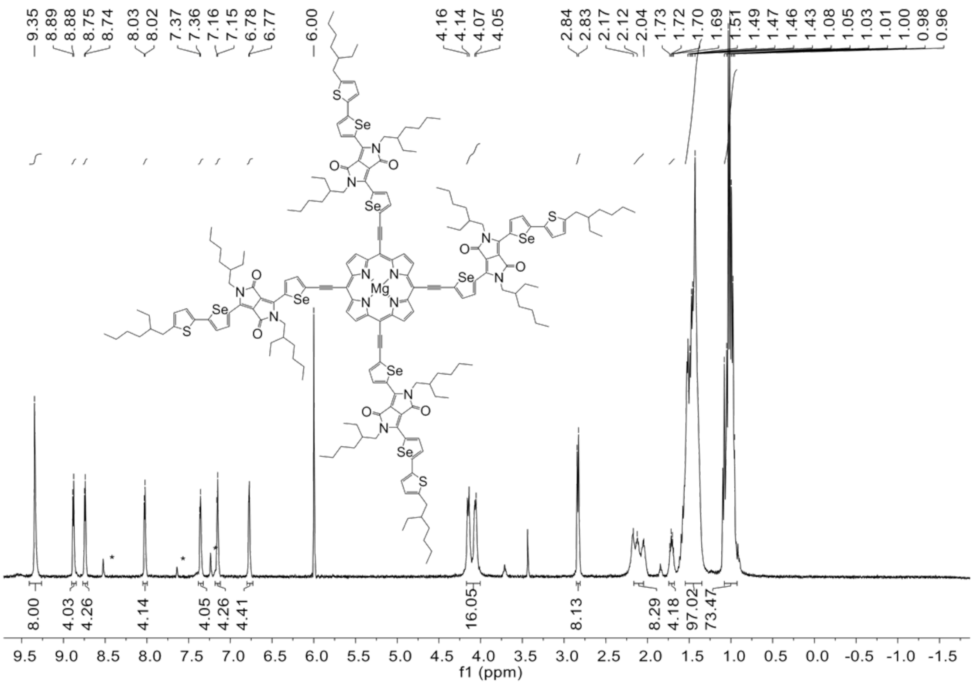

Figure S13. ^{1}H NMR spectrum for **2c**.

^{1}H NMR (400 MHz, tetrachloroethane-d_2 with 1% pyridine-d_5*, 100 °C): δ 9.35 (s, 8H, porphyrin), 8.88 (d, J = 4.2 Hz, 4H, selenophene), 8.74 (d, J = 4.0 Hz, 4H, selenophene), 8.03 (d, J = 4.1 Hz, 4H, selenophene), 7.36 (d, J = 4.0 Hz, 4H, selenophene), 7.16 (d, J = 3.4 Hz, 4H, thiophene), 6.77 (d, J = 2.6 Hz, 4H, thiophene), 4.11 (m, 16H, NCH_2), 2.83 (d, J = 6.7 Hz, 8H, thiophene-CH_2), 2.15 (m, 8H, CH), 1.71 (m, 4H, CH), 1.55–1.35 (m, 96H, CH_2), 1.08–0.96 (m, 72H, CH_3).

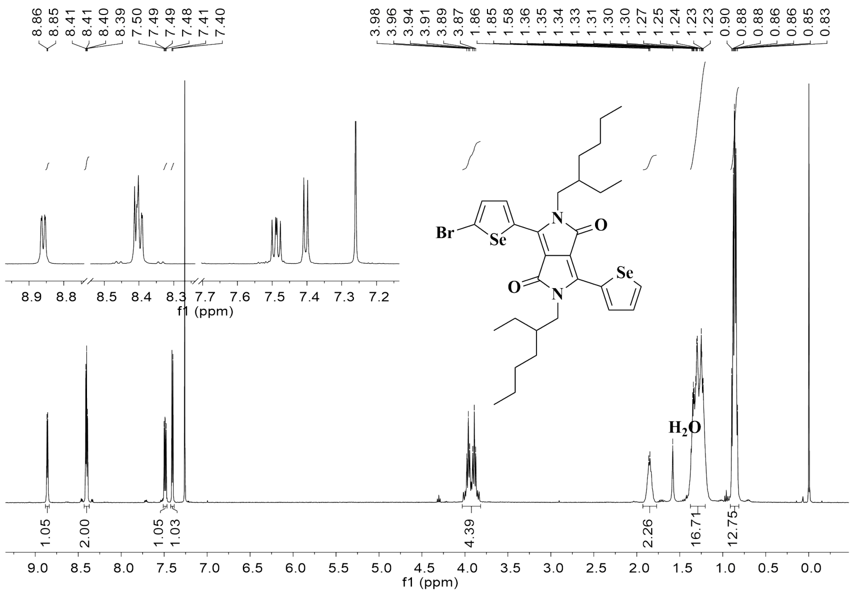

Figure S14.^{1}H NMR spectrum for **Br-Se-DPP**.

^{1}H NMR (400 MHz, $CDCl_3$): δ 8.86 (d, J = 3.2 Hz, 1H, selenophene), 8.40 (d, J = 3.0 Hz, 2H, selenophene), 7.49 (d, J = 4.1 Hz, 1H, selenophene), 7.40 (d, J = 4.4 Hz, 1H, selenophene), 3.98–3.91 (m, 4H, NCH_2), 1.85 (s, 2H, CH), 1.37–1.21 (m, 16H, CH_2), 0.90–0.83 (m, 12H, CH_3).

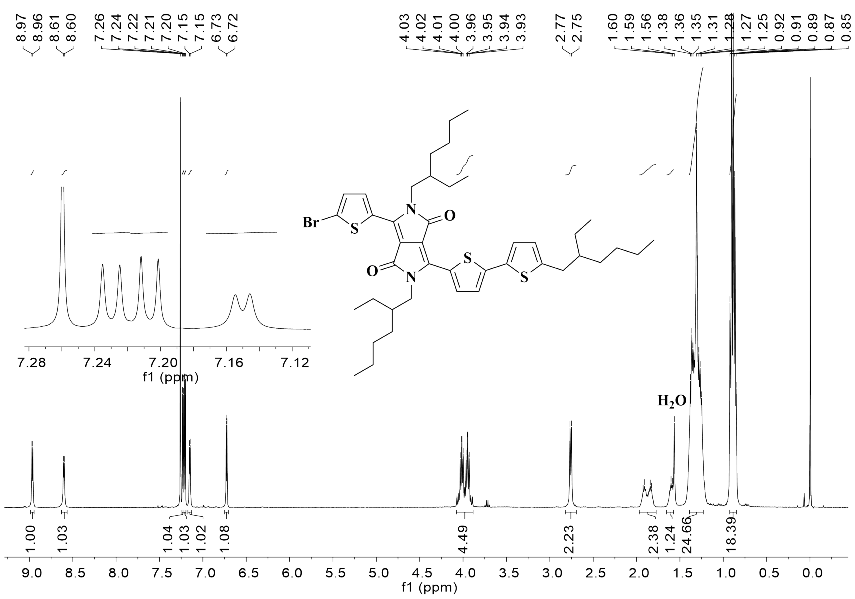

Figure S15. ^{1}H NMR spectrum for **Br-DPP-Th**.

^{1}H NMR (400 MHz, $CDCl_3$): δ 8.97 (d, J = 4.2 Hz, 1H, thiophene), 8.60 (d, J = 4.2 Hz, 1H, thiophene), 7.23 (d, J = 4.2 Hz, 1H, thiophene), 7.21 (d, J = 4.2 Hz, 1H, thiophene), 7.15 (d, J = 3.6 Hz, 1H, thiophene), 6.73 (d, J = 3.6 Hz, 1H, thiophene), 4.03–3.93 (m, 4H, NCH_2), 2.76 (d, J = 6.7 Hz, 2H, thiophene -CH_2), 1.88 (m, 2H, CH), 1.59 (m, 1H, CH), 1.39–1.22 (m, 24H, CH_2), 0.92–0.85 (m, 18H, CH_3).

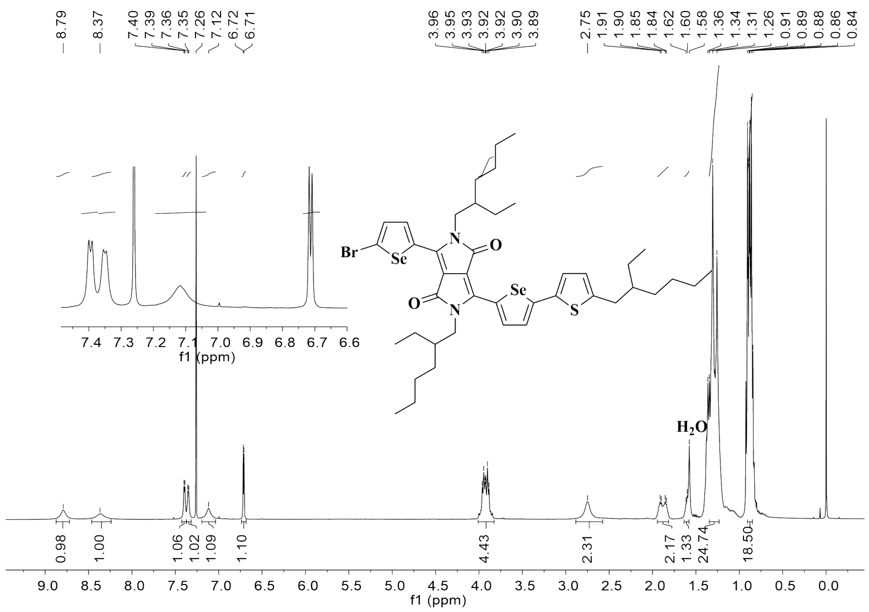

Figure S16. ^{1}H NMR spectrum for **Br-Se-DPP-Th**.
^{1}H NMR (400 MHz, $CDCl_3$): δ 8.79 (s, 1H, selenophene), 8.37 (s, 1H, selenophene), 7.39 (d, J = 4.3 Hz, 1H, selenophene), 7.35 (d, J = 4.3 Hz, 1H, selenophene), 7.12 (s, 1H, thiophene), 6.71 (d, J = 3.6 Hz, 1H, thiophene), 3.92 (m, 4H, N-CH_2), 2.75 (s, 2H, thiophene-CH_2), 1.95–1.82 (m, 2H, CH), 1.60 (m, 1H, CH), 1.41–1.18 (m, 24H, CH_2), 0.95–0.77 (m, 18H, CH_3).

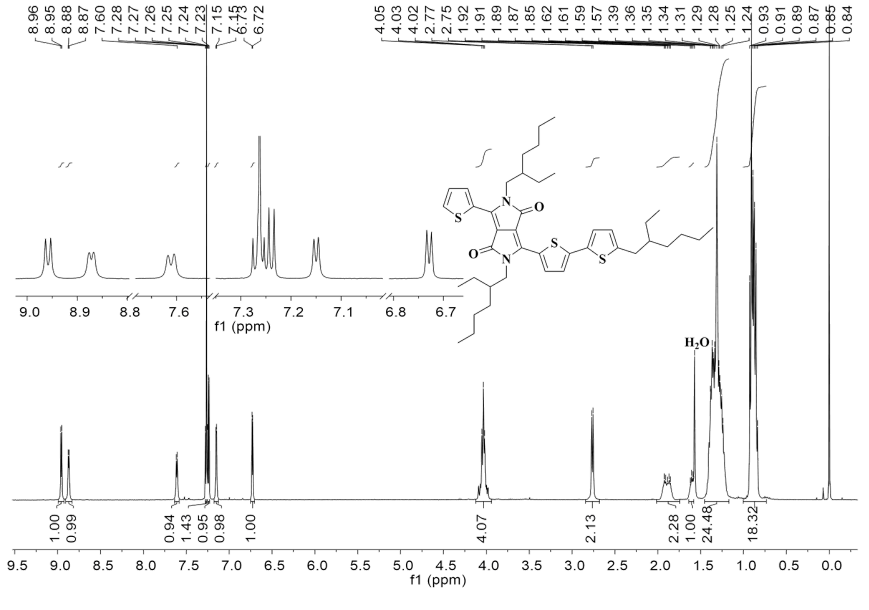

Figure S17. ^{1}H NMR spectrum for **S-DPP-Th**.
^{1}H NMR (400 MHz, $CDCl_3$): δ 8.96 (d, J = 4.2 Hz, 2H, thiophene), 8.87 (d, J = 3.9 Hz, 2H, thiophene), 7.29–7.25 (m, 1H, thiophene), 7.24 (d, J = 4.1 Hz, 1H, thiophene), 7.15 (d, J = 3.6 Hz, 1H, thiophene), 6.73 (d, J = 3.6 Hz, 1H, thiophene), 4.04 (m, 4H, N-CH_2), 2.76 (d, J =6.8 Hz, 2H, thiophene-CH_2), 2.00–1.77 (m, 1H, CH), 1.61 (m, 1H, CH), 1.40–1.24 (m, 24H, CH_2), 0.93–0.84 (m, 18H, CH_3).

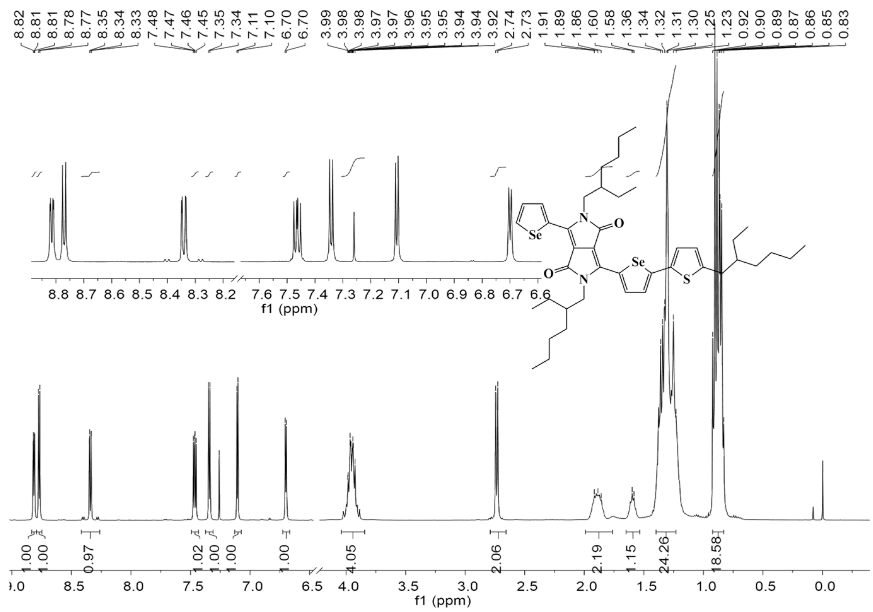

Figure S18. ^{1}H NMR spectrum for **Se -DPP-Th**.

^{1}H NMR (400 MHz, $CDCl_3$): δ 8.82 (d, J = 4.1 Hz, 1H, selenophene), 8.77 (d, J = 4.4 Hz, 1H, selenophene), 8.34 (d, J = 5.6 Hz, 1H, selenophene), 7.46 (d, J = 4.1 Hz, 1H, selenophene), 7.34 (d, J = 4.4 Hz, 1H, selenophene), 7.11 (d, J = 3.6 Hz, 1H, thiophene), 6.70 (d, J = 3.6 Hz, 1H, thiophene), 4.04–3.87 (m, 4H, N-CH_2), 2.74 (d, J = 6.8 Hz, 2H, thiophene-CH_2), 1.88 (m, 2H, CH), 1.59 (m, 1H, CH), 1.36–1.23 (m, 24H, CH_2), 0.92–0.83 (m, 18H, CH_3).

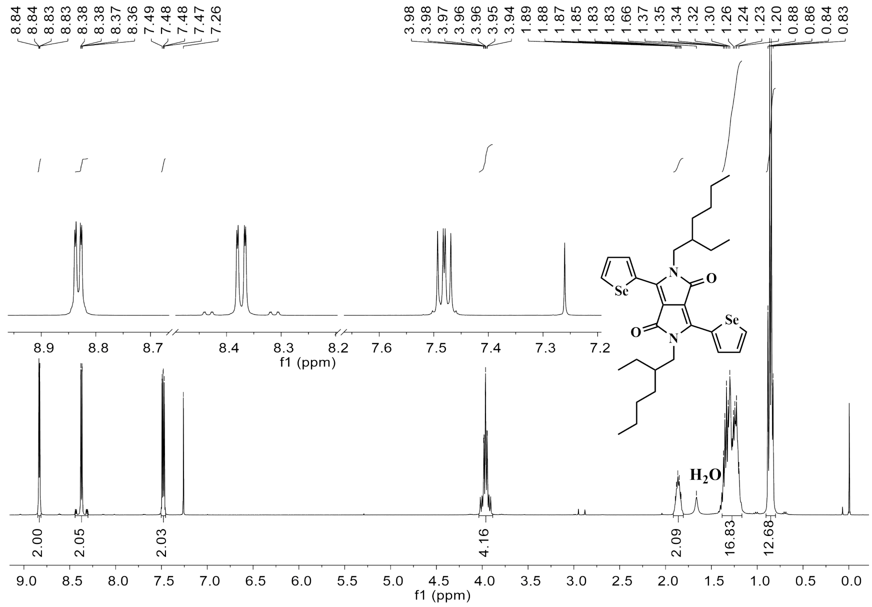

Figure S19. ^{1}H NMR spectrum for **Se-DPP**.

^{1}H NMR (400 MHz, $CDCl_3$): δ 8.84 (d, J = 4.1Hz, 1H, selenophene), 8.38 (d, J = 5.6 Hz, 1H, selenophene), 7.49 (d, J = 5.6 Hz, 1H, selenophene), 3.97 (m, 2H, N-CH_2), 1.90–1.82 (m, 1H, CH), 1.38–1.20 (m, 16H, CH_2), 0.97–0.83 (m, 12H, CH_3).

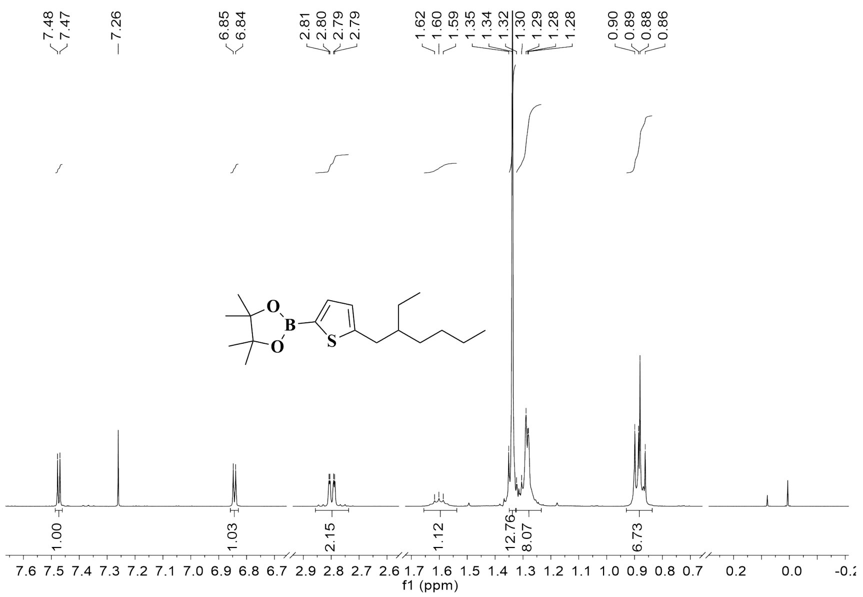

Figure S20. ^{1}H NMR spectrum for **Th-Bpin**.
^{1}H NMR (400 MHz, $CDCl_3$): δ 7.47 (d, J = 3.4 Hz, 1H, thiophene), 6.84 (d, J = 3.4 Hz, 1H, thiophene), 2.80 (d, J = 6.7 Hz, 2H), 1.63–1.53 (m, 1H, CH), 1.34 (s, 12H, Bpin), 1.29–1.28 (m, 9H, CH_2), 0.90–0.86 (m, 6H, CH_3).

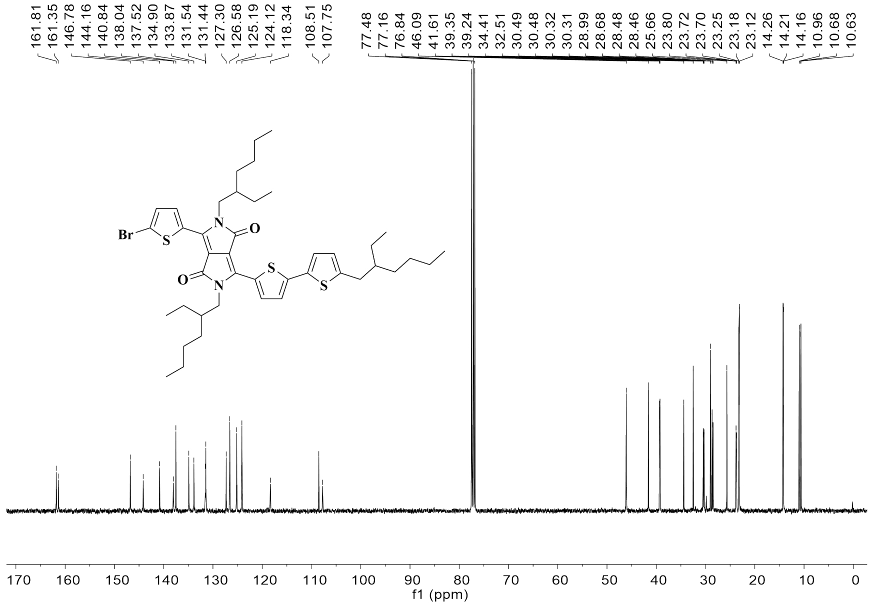

Figure S21. ^{13}C NMR spectrum for **Br-DPP-Th**.
^{13}C NMR (101 MHz, $CDCl_3$): δ 161.81, 161.35, 146.78, 144.16, 140.84, 138.04, 137.52, 134.90, 133.87, 131.54, 131.44, 127.30, 126.58, 125.19, 124.12, 118.34, 108.51, 107.75, 46.09, 41.61, 39.35, 39.24, 34.41, 32.51, 30.49, 30.48, 30.32, 30.31, 28.99, 28.68, 28.48, 28.46, 25.66, 23.80, 23.72, 23.70, 23.25, 23.18, 23.12, 14.26, 14.21, 14.16, 10.96, 10.68, 10.63.

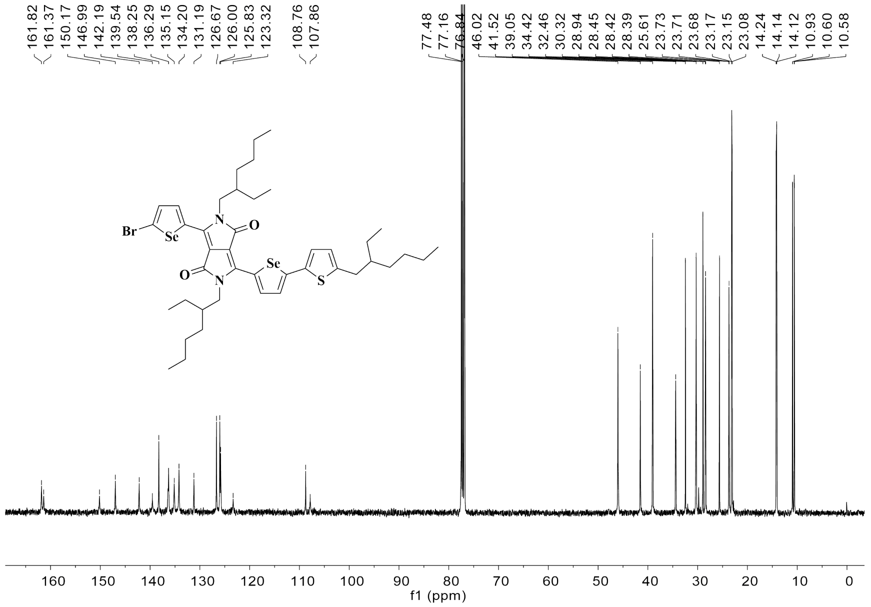

Figure S22. ^{13}C NMR spectrum for **Br-Se-DPP-Th**.
^{13}C NMR (101 MHz, $CDCl_3$): δ 161.82, 161.37, 150.17, 146.99, 142.19, 138.25, 136.29, 135.15, 134.20, 131.19, 126.67, 126.00, 125.83, 123.32, 108.76, 46.02, 41.52, 39.05, 34.42, 32.46, 30.32, 28.94, 28.45, 28.42, 28.39, 25.61, 23.73, 23.71, 23.68, 23.17, 23.15, 23.08, 14.24, 14.14, 14.12, 10.93, 10.60, 10.58.

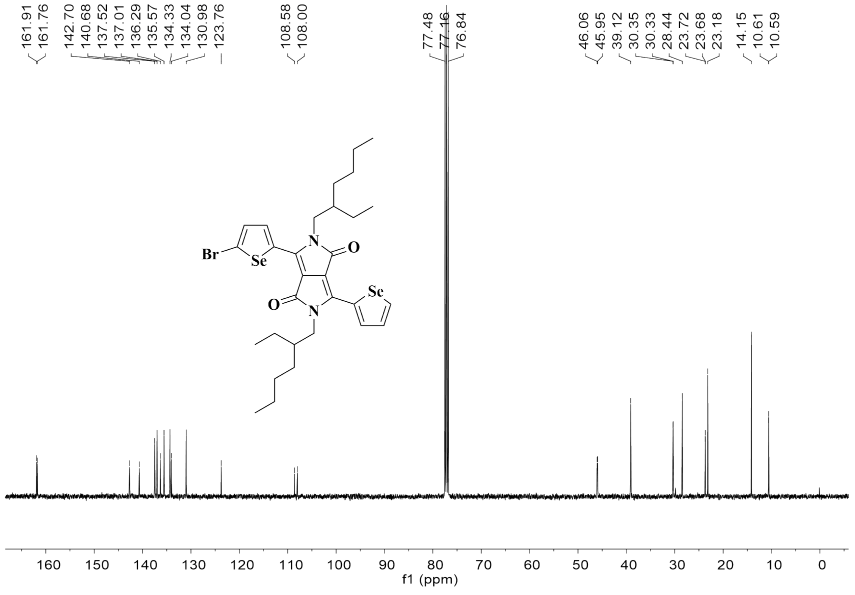

Figure S23. ^{13}C NMR spectrum for **Br-Se-DPP**.
^{13}C NMR (100 MHz, $CDCl_3$): δ 161.91, 161.76, 142.70, 140.68, 137.52, 137.01, 136.29, 135.57, 134.33, 134.04, 130.98, 123.76, 108.58, 108.00, 46.06, 45.95, 39.12, 30.35, 30.33, 28.44, 23.72, 23.68, 23.18, 14.15, 10.61, 10.59.

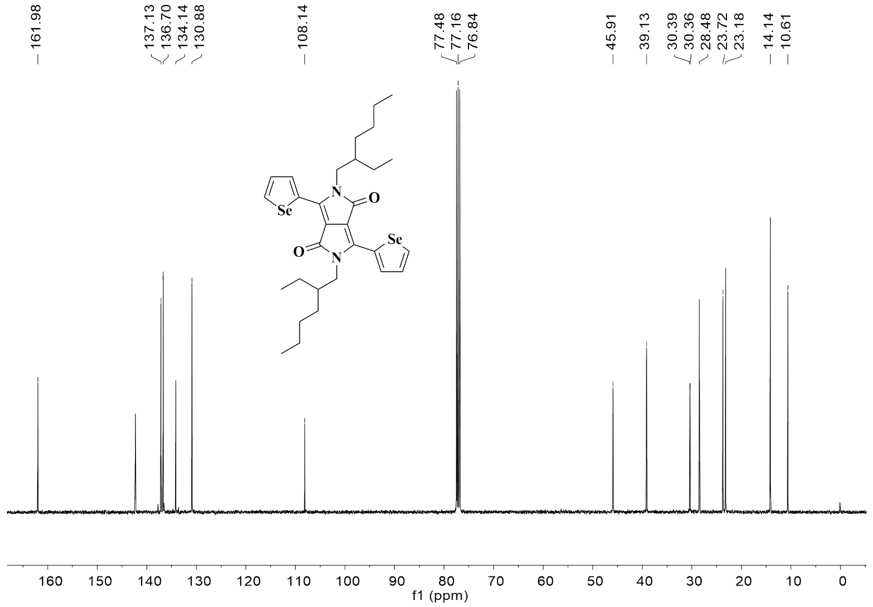

Figure S24. ^{13}C NMR spectrum for **Se-DPP**.
^{13}C NMR (100 MHz, $CDCl_3$): δ 161.98, 137.13, 136.70, 134.14, 130.88, 108.14, 45.91, 39.13, 30.39, 30.36, 28.48, 23.72, 23.18, 14.14, 10.61.

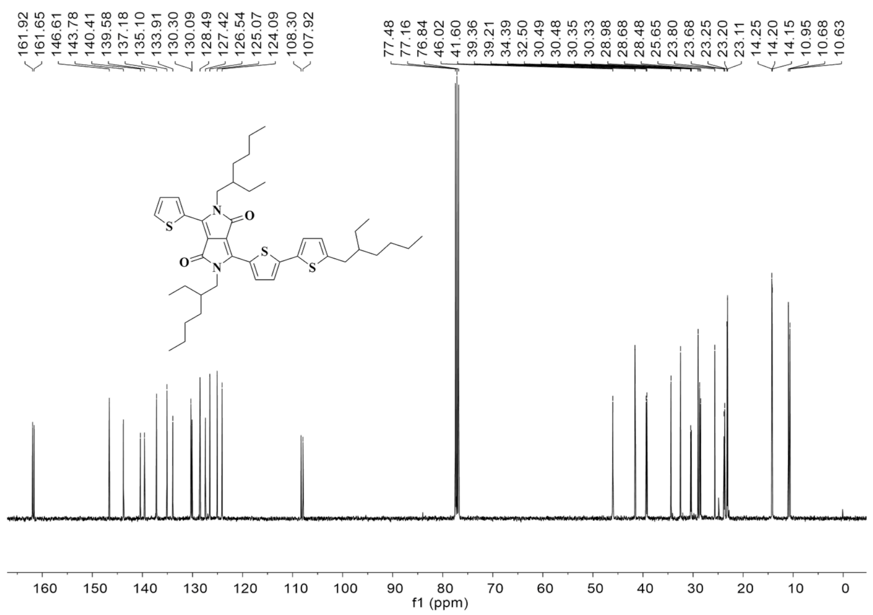

Figure S25. ^{13}C NMR spectrum for **DPP-Th**.
^{13}C NMR (100 MHz, $CDCl_3$): δ 161.92, 161.65, 146.61, 143.78, 140.41, 139.58, 137.18, 135.10, 133.91, 130.30, 130.09, 128.49, 127.42, 126.54, 125.07, 124.09, 108.30, 107.92, 46.02, 41.60, 39.36, 39.21, 34.39, 32.50, 30.49, 30.48, 30.35, 30.33, 28.98, 28.68, 28.48, 25.65, 23.80, 23.68, 23.25, 23.20, 23.11, 14.25, 14.20, 14.15, 10.95, 10.68, 10.63.

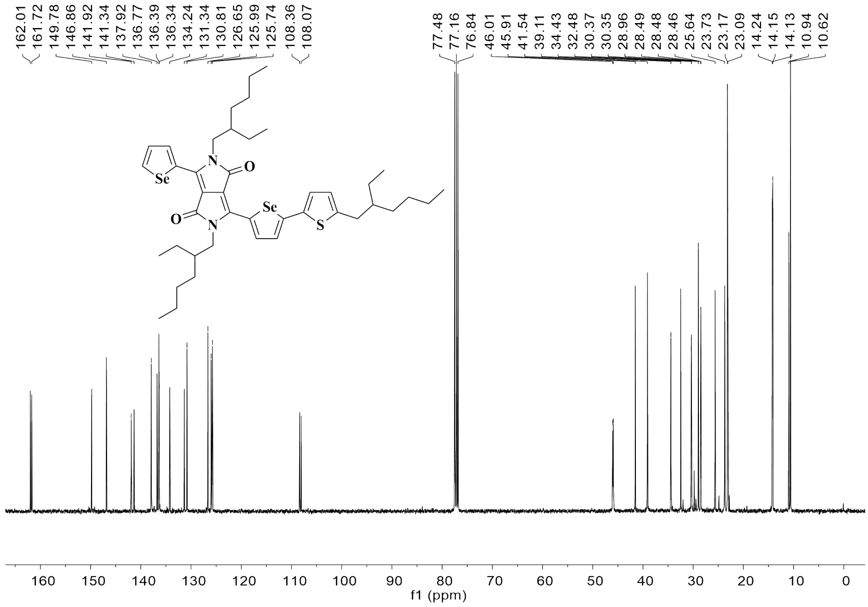

Figure S26. ^{13}C NMR spectrum for **Se-DPP-Th**.
^{13}C NMR (100 MHz, $CDCl_3$): δ 162.01, 161.72, 149.78, 146.86, 141.92, 141.34, 137.92, 136.77, 136.39, 136.34, 134.24, 131.34, 130.81, 126.65, 125.99, 125.74, 108.36, 108.07, 46.01, 45.91, 41.54, 39.11, 34.43, 32.48, 30.37, 30.35, 28.96, 28.49, 28.48, 28.46, 25.64, 23.73, 23.17, 23.09, 14.24, 14.15, 14.13, 10.94, 10.62.

8. MALDI-TOF HRMS Data

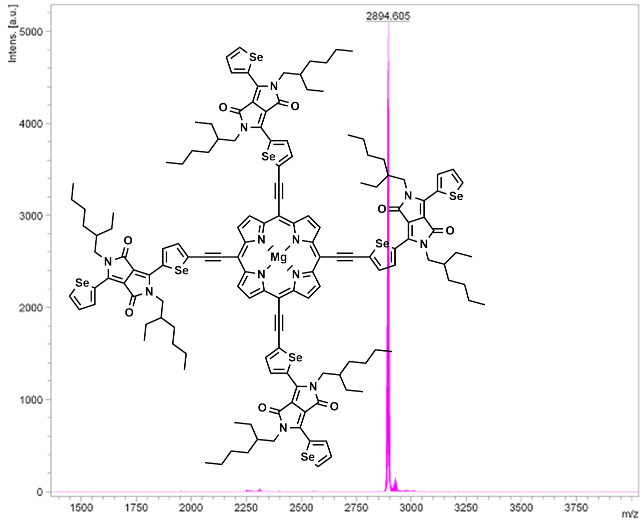

Figure S27. HRMS spectrum for **2a**. MALDI-TOF-HRMS (+) (*m*/*z*): calcd for $C_{148}H_{164}MgN_{12}O_8Se_8$ (M^+): 2894.6070, found 2894.6050.

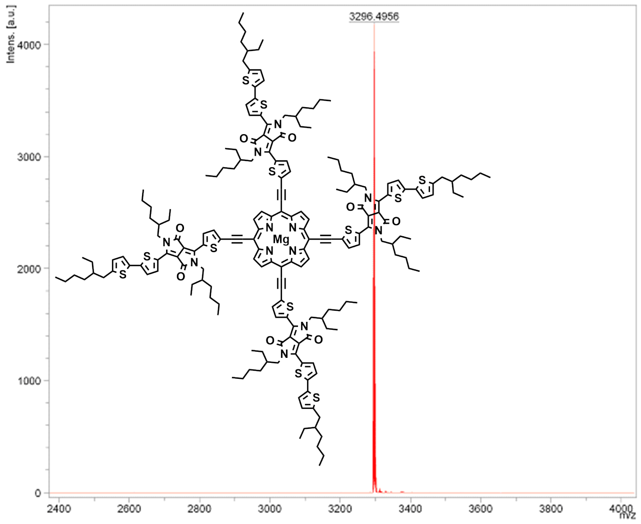

Figure S28. HRMS spectrum for **2b**. MALDI-TOF-HRMS (+) (*m*/*z*): calcd for $C_{196}H_{236}MgN_{12}O_8S_{12}$ (M^+): 3296.4972, found 3296.4956.

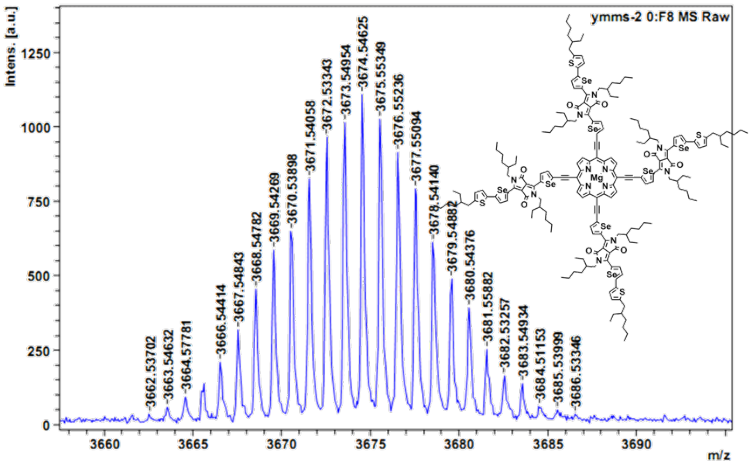

Figure S29. HRMS spectrum for **2c**. MALDI-TOF-HRMS (+) (*m*/*z*): calcd for $C_{196}H_{236}MgN_{12}O_8S_4Se_8$ (M^+): 3677.0484, found 3677.5509.

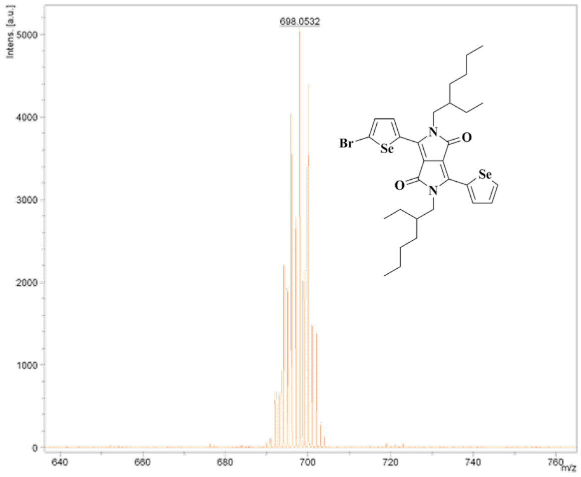

Figure S30. HRMS spectrum for **Br-Se-DPP**. MALDI-TOF-HRMS (+) (*m*/*z*): calcd for $C_{30}H_{39}BrN_2O_2Se_2$ (M^+): 698.0525, found 698.0532.

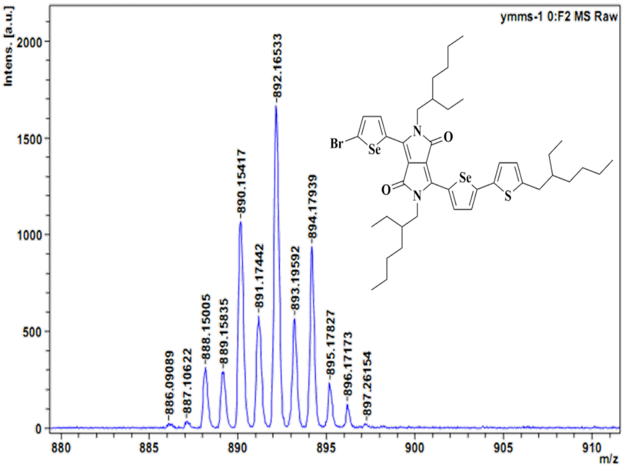

Figure S31. HRMS spectrum for **Br-Se-DPP-Th**. MALDI-TOF-HRMS (+) (*m*/*z*): calcd for $C_{42}H_{57}BrN_2O_2SSe_2$ (M^+): 892.1655, found 892.1653.

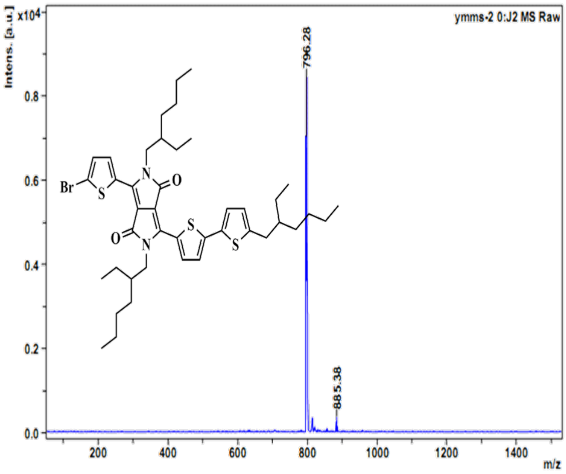

Figure S32. HRMS spectrum for **Br-DPP-Th**. MALDI-TOF-HRMS (+) (*m*/*z*): calcd for $C_{42}H_{57}BrN_2O_2S_3$ (M^+): 796.2766, found 796.2800.

References

1. D. Sahu, C.-H. Tsai, H.-Y. Wei, K.-C. Ho, F.-C. Chang and C. W. Chu, *J. Mater. Chem.*, 2012, **22**, 7945.
2. L. T. Dou, W. H. Chang, J. Gao, C. C. Chen, J. B. You and Y. Yang, *Adv. Mater.*, 2013, **25**, 825.
3. M. Shahid, T. McCarthy-Ward, J. Labram, S. Rossbauer, E. B. Domingo, S. E. Watkins, N. Stingelin, T. D. Anthopoulos and M. Heeney, *Chem. Sci.*, 2012, **3**, 181.
4. Z. Yi, L. Ma, B. Chen, D. Chen, X. Chen, J. Qin, X. Zhan, Y. Liu, W. Ong and J. Li, *Chem. Mater.*, 2013, **25**, 4290−4296.
